# Metal–organic frameworks and their composites for fuel and chemical production *via* CO_2_ conversion and water splitting

**DOI:** 10.1039/d1ra09063a

**Published:** 2022-04-14

**Authors:** Karanika Sonowal, Lakshi Saikia

**Affiliations:** Advanced Materials Group, Materials Sciences and Technology Division, CSIR-North-East Institute of Science & Technology Jorhat Assam-785006 India l.saikia@gmail.com lsaikia@neist.res.in; Academy of Scientific and Innovative Research (AcSIR) Ghaziabad-201002 UP India

## Abstract

Increase in the global energy demand has been leading to major energy crises in recent years. The use of excess fossil fuels for energy production is causing severe global warming, as well as energy shortage. To overcome the global energy crisis, the design of various chemical structures as efficient models for the generation of renewable energy fuels is very much crucial, and will limit the use of fossil fuels. Current challenges involve the design of Metal–Organic Framework (MOF) materials for this purpose to diminish the energy shortage. The large surface area, tunable pore environment, unique structural property and semiconducting nature of the highly porous MOF materials enhance their potential applications towards the production of enhanced energy fuels. This review is focused on the architecture of MOFs and their composites for fuels and essential chemicals production like hydrogen, methane, ethanol, methanol, acetic acid, and carbon monoxide, which can be used as renewable fuel energy sources to limit the use of fossil fuels, thereby reducing global warming.

## Abstract 2

This review is focused on the application of MOFs and their composites for the production of fuels and chemicals like hydrogen, methane, ethanol, methanol, acetic acid, and carbon monoxide, which can be used as renewable energy sources to limit the use of fossil fuels and thereby reduce global warming.

## Graphical abstract

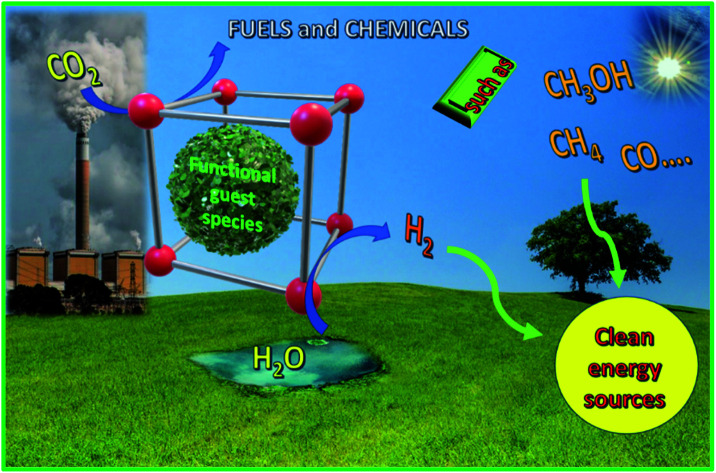

## 1. Introduction

Considering the current energy demand, fossil fuels are used on a large scale mostly for electricity production to fulfill the global energy requirements. The burning of fossil fuels releases large amounts of greenhouse gases including carbon dioxide (CO_2_) into the atmosphere, which causes global warming. CO_2_ absorbs energy from infrared radiation for a longer time and releases it back into the air, thereby increasing the global temperature. As a consequence, various disasters are occurring, such as the melting of glaciers, floods, and the rise in sea levels, making the world an unsuitable place to survive for all living beings, as well as to the environment. The use of excess fossil fuels to accomplish energy requirements leads to the energy crises. It awakens researchers to come up with alternate energy sources to satisfy the energy demand. Therefore, challenges arise in the design of different models to produce efficient energy sources with no environmental drawbacks. Metal–organic framework (MOF)-based structures can be considered an effective model to produce renewable energy sources, photocatalytically, electrocatalytically and photoelectrocatalytically. There are some reports on MOF-based materials that mention their potential applications in fuel cells, lithium-ion batteries, gas tanks, solar cells and supercapacitors.^1–3^ There are many reports on energy storage and conversion applications using MOF-based materials.^4–6^ Scientists are trying to solve the energy and environmental issues in many ways. The structural diversity, uniform porosity, host–guest interactive property, tunability, functionalization and semiconducting behavior of MOFs stimulate the researchers to use MOF-based materials for energy storage and conversion applications. Furthermore, these unique properties of MOFs make them superior to other inorganic porous materials. The presence of inorganic metal ions and photoactive-functionalized organic ligands in the MOFs framework enhances their potential to act as catalytically active surface sites for promoting chemical reactions in the presence of light sources to boost energy applications.^1^ The integration of various metals, metal oxides, phosphides, sulphides, quantum dots and carbon based materials is now becoming a very challenging topic in the architecture of metal–organic framework composites for various applications.^2,7,8^ The incorporation of electron efficient guest molecules into MOFs has a greater impact on many kinds of applications. The excellent gas adsorption and storage ability of MOFs signify their potential uses in fuel cells, vehicle gas reservoirs and others.^1^ The development in nanotechnology and proper characterization equipment helps to design and characterize new materials in a proper way to enhance energy resources.

This review is based on the study of previous reports in the design of various MOFs and their composites for the development of renewable energy resources to boost the global economy. Different MOF-based materials with different synthetic methodology and advantages/disadvantages for energy related applications like fuel production have been discussed in this article. Specifically, sources of fuel energy from the CO_2_ conversion into value-added chemicals and fuels, and water splitting reactions for hydrogen and peroxide production are discussed in this article. Through this, we aim to provide an overall summary of the current ongoing research using MOF-based materials for energy applications, showcasing its importance to the scientific community, while encouraging more contributions to this field to improve the global economy. We hope that this article will help researchers exploit MOFs-based materials for energy applications.

### 1.1. MOFs and their composites

**Fig. 1:**
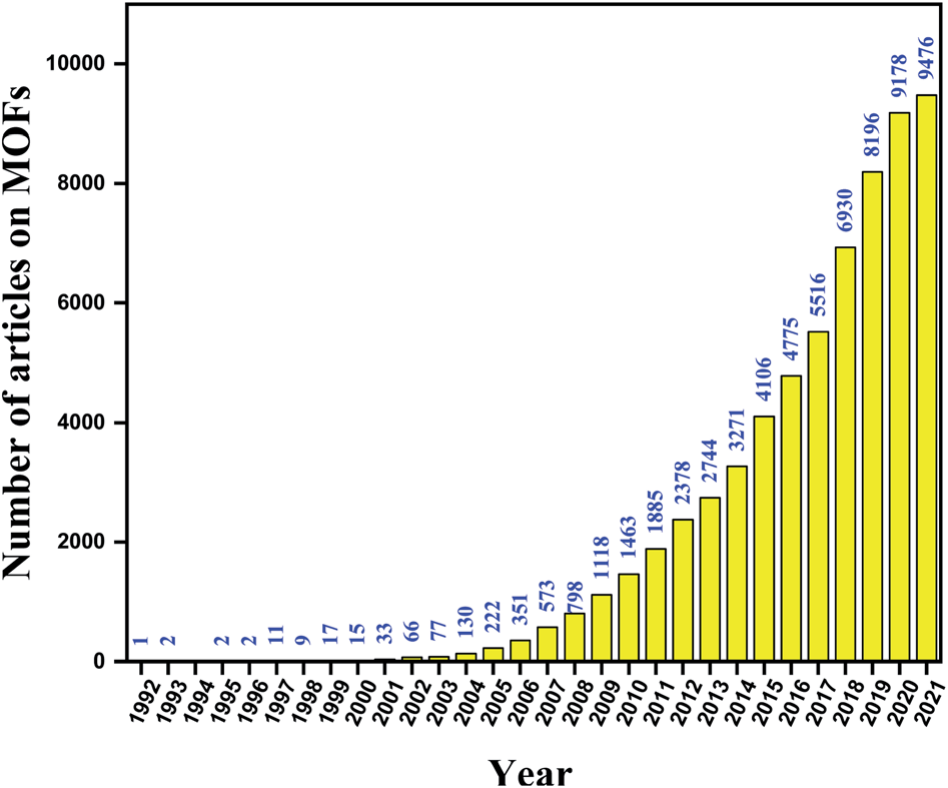
Flow chart representing different MOFs in various applications since its origin (data collected from Web of Science database).

There are challenges in the architecture of MOFs and their composite materials that exhibit potential applications in various fields, including energy-related applications. Metal–organic frameworks (MOFs) are a class of porous materials with strong coordination geometry, which are formed by the connection of metal ions or clusters with organic ligands or linkers in an ordered way. These are highly crystalline materials with ordered porosities. MOFs can exhibit one-, two- and three-dimensional framework structures with large surface area ranging from 1000 to 10 000 m^2^ g^−1^.^9–12^ The surface area for MOFs is larger than traditional porous materials like zeolites and other carbon-based materials. Moreover, MOFs exhibit unique structural diversity, tunable functionality, uniform porosity, flexibility, and multifunctionality, which makes them superior to other porous materials. MOFs were pioneered by Omar Yaghi in the late 1990s. Since then, more than 90 000 varieties of MOFs have been reported. There are a large number of applications using MOFs in gas adsorption, gas separation, catalysis, bioimaging, drug delivery, sensing, and energy applications that have been reported so far.^13–20^ Moreover, MOFs with controlled morphology and size open up a new way to traditional pristine MOFs. MOFs also act as support substrates to accommodate metals, metal-oxides/sulfides/phosphides, nanoparticles, and other complexes to form different nanostructures. The unique structural properties of MOFs have drawn major attention from young chemists and scientists to explore this field. A flow chart of different MOFs on various applications in each decade from its origin is exhibited in Fig. 1.

**Fig. 2:**
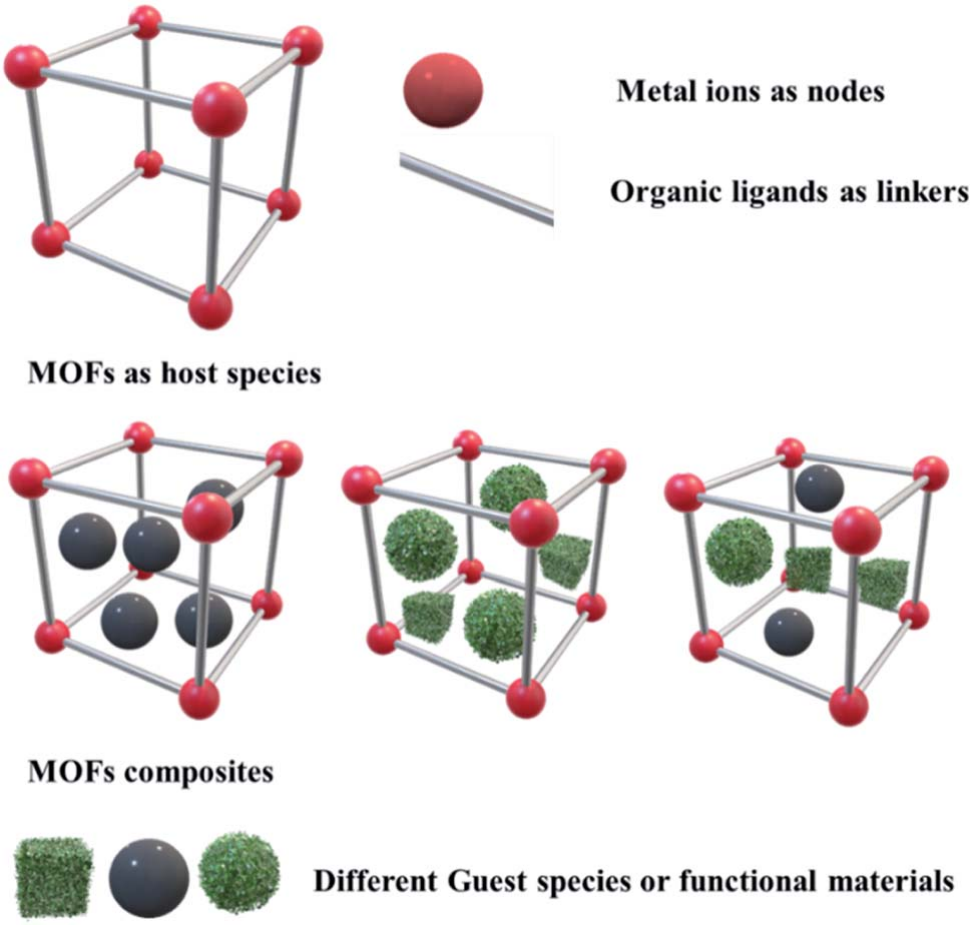
Schematic representation of MOFs and their different composites.

Post-synthetic modifications and formulating different composites of MOFs have resulted in enhanced activities of MOF-based materials, which are now becoming very challenging for the researchers to develop newly designed MOFs and methods by modifications. Due to the large surface area and uniform pore volume, MOFs can act as host materials to welcome guest molecules like nanoparticles, quantum dots, metals and its oxides, sulfides, and carbon-based materials like graphene, graphitic carbon nitride, nanotubes, nanorods and other functional materials to form different MOFs composites for enriching their activities in various fields.^21,22^ The integration of these guest materials into MOFs causes changes in the chemical, physical and electronic properties of the composite materials, which result in better activity than the parent MOFs. Along with this, introducing co-catalysts into porous MOFs slow down the recombination rate of photogenerated charge carriers, enriching the reaction rates and providing stability to the system. In most cases, the incorporation of different functional materials leads to enhanced activities. Therefore, the syntheses of different MOFs composites have become a topic of interest nowadays. Considering the major energy crisis and environmental issues, scientists have shown great interest in working in energy applications using MOF-based materials owing to the budding research in fuel cells, supercapacitors, photocatalytic hydrogen evolution and CO_2_ reduction into useful chemicals and fuels in recent years (Fig. 2).^2,5^

There are several methods for synthesizing MOFs and their composites, such as solvothermal, hydrothermal, mechanochemical, microwave synthesis, son-chemical, microemulsion, diffusion, template strategy and electrochemical methods, which have been reported so far.^23^ Among these, solvothermal and hydrothermal methods have been considered the most prevailing methods for the synthesis of MOF-based materials. Other synthetic methods are also considered as effective ones, which provide nanoscale controllable MOFs crystals in short reaction times. The synthesis of MOF-based materials depends on several parameters like the temperature, pressure, reaction time, metal nodes, ligands, presence of counterions and reaction kinetics that leads to nucleation and crystal growth.^24^ Solvent selection is one of the most important things for any kind of reaction, as it is a key to determine the activation energy and reaction thermodynamics. In addition, the single crystal growth in MOFs is of utmost importance as many of us face difficulties to do so. Furthermore, single crystals are highly ordered structures with unique thermal, optical, mechanical, electrical and other superior properties than polycrystalline materials. Owing to the excellent optical and electrical properties of single crystals, they are utilized in various important applications such as wireless and satellite communication, light-emitting diodes (LEDs), electronics, photodetectors, and wide band-gap devices. With the developing technology, demands for single crystals are growing accordingly. The growth of single crystals with high quality is a very challenging topic since the last decade. From the literature, it has been found that there are three general approaches for the growth of bulk inorganic single crystals: melt, solution and vapor phase.^25^ Single crystal growth from melt is the most common way used for single crystal growth *via* solidification and crystallization of the melted material. This method allows for the growth of large single crystals of excellent quality in a short period of time compared to other methods.^26^ However, this method also has some limitations, such as difficulty in the maintenance of a stable temperature, gaining high melting points of some materials, and obtaining chemical consistency. Solution-growth technique is another approach where single crystals are obtained by dissolving the material into a suitable solvent or flux.^27^ Among the solution-growth techniques, high-temperature solution growth or flux-growth is one of the most promising techniques for materials that incongruently melt, and can be applied for single crystal growth of the materials that cannot be processed by the melt growth process. The advantage of using this technique is that the single crystals of materials can be obtained below their melting temperatures. This is a slow process of crystal growth, and the existence of flux ions is inescapable in this method. Another method for single crystal growth is the vapor-phase growth *via* sublimation process at low temperature. This lower temperature enhances the crystal quality, avoiding impurities due to phase transitions. This method is used to obtain thin single crystal films rather than obtaining bulk single crystals. However, this method has some limitations, such as low crystal growth and low vapor transport rates, for which this method is less utilized as compared to the other two methods. In spite of the three conventional methods mentioned above, there is one more technique known as solid-state single crystal growth (SSCG) that has received much attention recently.^28–30^ This is a method *via* the solid-state conversion of polycrystalline materials into a single crystal. The SSCG method overcomes all of the limitations found in the conventional methods. Control of the microstructure development during the conversion of polycrystalline materials into single crystals is the most challenging part of this method, for which it is limited to a few systems.

In this article, different MOFs and their composites for fuel production applications have been discussed based on previous reports.

## 2. MOF-BASED materials for fuels production

### 2.1. CO_2_ conversion

**Fig. 3:**
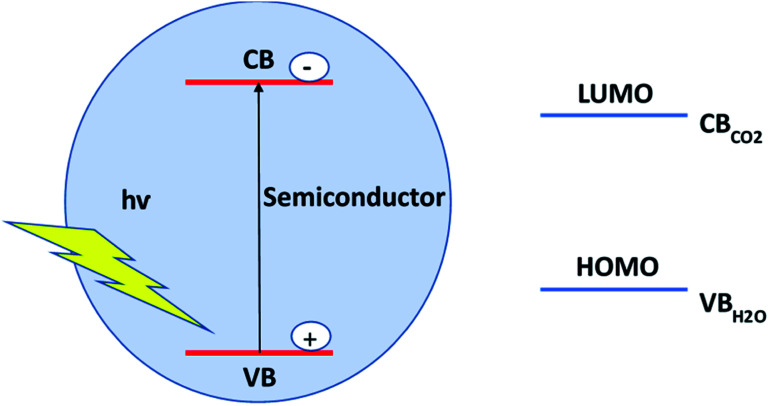
Required band positions for the CO_2_ reduction.

Global warming and the shortage of renewable energy resources are a major concern worldwide for the future energy supply and solving environmental issues. In view of the natural photosynthesis process where solar energy is converted into chemical energy, scientists are trying to develop alternate processes by means of the artificial photosynthesis method where CO_2_ reduction will produce hydrocarbons to fulfill the energy requirements. Several efforts have been made to develop appropriate catalysts for CO_2_ storage and conversion. Still, challenges remain for developing excellent materials for CO_2_ storage and reduction into useful compounds and fuels, as not all types of catalysts are capable of giving the desired products. This leads to various difficulties of CO_2_ reduction. These include high overpotential of CO_2_ (−1.9 V *vs.* SHE), less selectivity for product formation, low reaction rate, and the poor solubility of CO_2_.^31^ In addition, the band gap energy of semiconductor catalysts is another important factor for CO_2_ reduction. For a spontaneous CO_2_ reduction process, the conduction band position of the semiconductor should be higher or more negative than the redox potential for CO_2_ reduction. Furthermore, the valence band position of the semiconductor should be lower or more positive than the redox potential for water oxidation into oxygen molecule, as shown in Fig. 3. Moreover, the reaction medium and reaction conditions such as temperature, pressure play important roles for CO_2_ reduction. For efficient production of carbonaceous products from CO_2_, the chemisorption of CO_2_ on the catalytically active sites of the catalyst surface is very important for activating the CO_2_ molecule. To chemisorb CO_2_ on the catalyst surface, proper dissolution of CO_2_ in the solvent is another important factor. Since CO_2_ shows poor solubility in the water medium, it leads to a low reaction rate of CO_2_ reduction. To achieve the enhancement of CO_2_ adsorption, researchers have found alternate ways such as the use of non-aqueous solvents (DMSO, acetonitrile, ethyl acetate, DMF) and a mixture of aqueous/non-aqueous solvents as the reaction medium, and the design of catalysts with large catalytically active sites.^5^ It has been found that the use of sacrificial materials such as triethanolamine (TEOA), triethyl amine (TEA), ethanol, and water can improve the catalytic activity for CO_2_ conversion. CO_2_ reduction leads to various useful chemicals and fuels like HCOOH, CH_3_COOH, CH_4_, C_2_H_6_, CH_3_OH, and C_2_H_5_OH, which are considered as energy carriers or fuels and have industrial applications. The multifunctionality and tunable functionality of MOFs make them promising catalysts for CO_2_ storage and reduction activity. There are various reports of CO_2_ conversion into value-added chemicals and fuels using solar and electrical energy for MOFs-based materials.^32–35^ Still, challenges remain for the large-scale fuel production rate using MOFs-based materials for industrial applications.

Fuels like methane, methanol, and acetic acid have tremendous usefulness in industry and as renewable energy resources. To limit the use of fossil fuels as energy sources, it would be benign to be used non-fossil fuels as energy sources to protect the energy for future generations. Methane is used as a fuel in home accessories such as ovens, water heaters, automobiles, and turbines. Refined liquid methane with the combination of liquid oxygen can be used as a rocket fuel. Methane gas is utilized to run or power engines and turbines in factories. Industries like food processors, pulp and paper, petroleum refineries and companies that work with clay, stone and glass utilize the energy it discharges. Methanol is used as non-fossil fuel energy sources in combustion engines of vehicles, or it can be mixed with gasoline to produce an efficient fuel like octane with lower emissions than the typically used gasoline. Methanol can also be used in industries, and as a fuel in cooking and thermal applications. Acetic acid is used in industries like food, chemical, pharmaceutical, chemical and plastic. It can also be used as a fuel to generate electricity *via* SOFC technology, which can be considered the most promising method for the generation of electricity for future power sustainability. Similarly, some more non-fossil fuels can be generated by the CO_2_ conversion process, which have valuable uses in various aspects, including power generation.

Recent studies suggest three approaches for CO_2_ reduction (a) photocatalytic approach, (b) electrocatalytic approach, and (c) photoelectrocatalytic approach. The photocatalytic CO_2_ reduction is facilitated using solar energy and various powerful light sources like the Xe lamp and Hg lamp at particular wavelengths. The electrocatalytic process involves using electrical energy into useful products. The photoelectrocatalytic approach involves using both light energy and electrical energy sources. In the photoelectrochemical process, the use of light energy sources reduces the electricity consumption of the applied external voltage, and the application of an external voltage helps to separate the photogenerated electron–hole pairs, thereby enhancing the catalytic efficiency. Furthermore, the use of half cells inhibits the reoxidation of active final products. All of these three approaches are very important to activate CO_2_ reduction. Still, the photocatalytic approach seems to be an ideal way for CO_2_ reduction due to high abundance of solar energy. Moreover, some drawbacks have been found for this process, such as the fast charge recombination rate and difficulty in generating a large number of catalytically active sites.

#### (a) Photocatalytic approach

MOFs with tailored hybrid inorganic–organic building blocks and large surface areas with uniform porosity enhance the CO_2_ conversion rate. This is achieved by generating catalytically active sites on its surface and by transferring electrons from these catalytically active sites to activate the CO_2_ molecule for the production of value-added chemicals and fuels. To activate the CO_2_ molecule, the proper solubility of CO_2_ in water is an important factor. Otherwise, it will become a major drawback for CO_2_ conversion. There are some approaches to enhance CO_2_ dissolution: the use of appropriate non-aqueous solutions, the suitable design of photocatalysts, the increase of the CO_2_ partial pressure and having a high CO_2_ flow rate. The photocatalytic CO_2_ conversion depends mainly on three factors: (a) photoreactor design, (b) photocatalyst used, and (c) experimental parameters (temperature, pressure, solvent, light source, wavelength, catalyst concentration).

Lin *et al.* first reported a doped UiO-67 metal–organic framework for photochemical CO_2_ reduction using visible light by incorporation of Re^I^(CO)_3_(dcbpy)Cl(H_2_L_4_) into the UiO-67 framework.^36^ The photocatalytic CO_2_ reduction activity into CO was studied using CO_2_-saturated with acetonitrile (MeCN) and triethylamine as a sacrificial reducing agent (MeCN : Triethylamine = 20 : 1). The [Re^I^(CO)_3_(dcbpy)Cl]-doped UiO-67 showed excellent CO_2_ reduction compared to the homogeneous catalyst, [Re^I^(CO)_3_(dcbpy)Cl]. The reduction process follows a unimolecular mechanism because of the uniform dispersion of the catalyst into MOFs-active surface sites [Re^I^(CO)_3_(dcbpy)Cl] (Re ∼50%) doping in MOF enhanced the photochemical CO_2_ reduction activity.

**Fig. 4:**
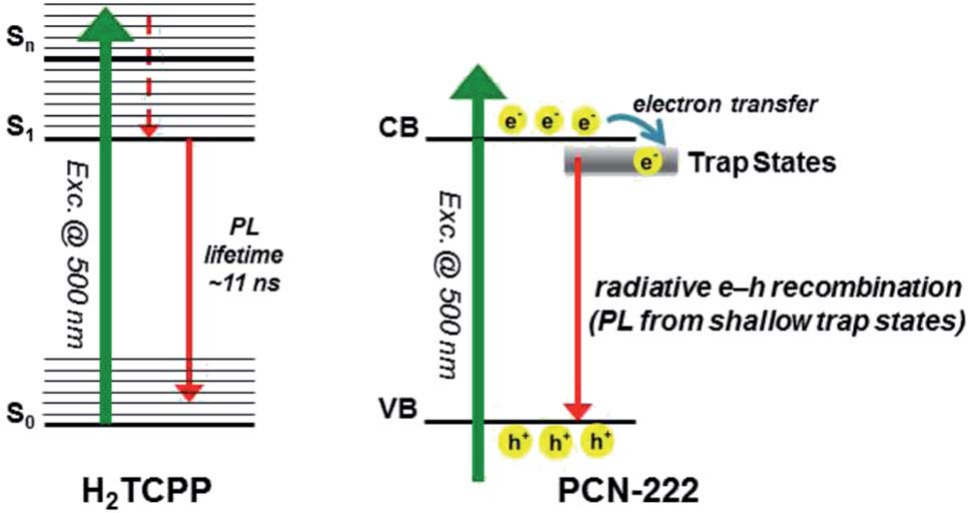
Mechanisms for the photoexcited dynamics involved in H_2_TCPP (left) and PCN-222 (right). Left side: S_0_, S_1_, and S_*n*_ denote the electronic ground state, the first electronically excited state, and a certain high-lying electronically excited state reached by the 500 nm photon, respectively. Right side: conduction band (CB) and valence band (VB). This figure has been adapted from ref. 37 with permission from the AMERICAN CHEMICAL SOCIETY, copyright 2015.

Jiang *et al.* reported on one highly photoactive porphyrin based-Zirconium MOF catalyst, PCN-222 for the photocatalytic CO_2_ reduction into a formate anion using solar energy.^37^ Triethanolamine (TEOA) was used as a sacrificial agent. Ultrafast spectroscopy with time-resolved photoluminescence spectroscopy unveiled that presence of a long-term electron trap state in the PCN-222 (Zr ∼2%) catalyst enhanced the photocatalytic conversion activity by inhibiting the electron–hole pair recombination and enhancing the reaction time. It provided greater activity to the PCN-222 catalyst than the H_2_TCPP ligand present therein. The photocatalytic activity was studied using 50 mg photocatalyst in MeCN/TEOA (10 : 1 v/v, 60 mL) solution, and the solution was irradiated using a Xe lamp (wavelength ∼420–800 nm). The spontaneous HCOO^−^ anion production rate was found to increase by up to 30 μmol in 10 h, which was much higher as compared to previous reports of MIL-125-NH_2_ and UiO-66-NH_2_ under similar conditions.^38^ This work confirms that the presence of an electron trap state in a catalyst can improve the catalytic efficiency (Fig. 4).

Choi and his co-workers reported on one nanoparticle-coated MOF photocatalyst for enhanced CO_2_ reduction to CO.^39^ The Re_*n*_-MOF photocatalyst contained two different functional units for the enhancement of the catalytic activity and catalytic stability, which were combined as a single construct. The highest photocatalytic activity was observed for Re_3_-MOF by controlling the density of the framework, where *n* = 0, 1, 2, 3, 5, 11, 16, and 24 complexes per unit cell. They confirmed that the equilibrium vicinity between the photoactive centers enhances the photocatalytic activity. Based on this theory, they coated Re_3_-MOF (Re ∼23%) into Ag nanocubes (Ag ⊂Re_3_-MOF), which resulted in a seven-fold enhancement of the CO_2_ conversion to CO under visible light irradiation. The photocatalytic experiments were studied using a sealed batch-type custom cell by dispersing the photocatalyst in a CO_2_ saturated solution of acetonitrile/triethylamine, where triethylamine was used as a sacrificial electron donor. The solution was irradiated using Xe lamp (300 W) with visible band-pass filters in a wavelength range of 400 to 700 nm.

As Cadmium sulfide (CdS) has excellent visible-light responsive behavior and a suitably positioned conduction band for the photocatalytic reduction of CO_2_, there are still some limitations when using CdS as semiconducting photocatalysts due to the fast recombination of photogenerated electron–hole pairs, photo corrosion, lack of catalytic sites and low CO_2_ absorption.^40^ Recent progress has reported that CdS with MOFs combination can overcome these drawbacks by increasing the visible light response.^41–43^ However, limitations arise for the CdS@MOFs composites for the efficient photocatalytic CO_2_ reduction activity due to the lack of catalytic sites and the bigger size of CdS.^44^ Han *et al.* reported on the ternary CdS/UiO-bpy/Co composites, where inorganic semiconductors and molecular redox catalysts were incorporated through UiO-bpy MOFs for the first time to see the photocatalytic CO_2_ conversion activity under visible light.^45^ The ternary composites exhibited an excellent CO production rate, 235 μmol g^−1^ h^−1^ in 10 h irradiation, which was a 10.2-fold improvement in the CO production rate compared to the parent CdS (CO, 23 μmol g^−1^ h^−1^) and CdS/UiO-bpy (CO, 0 μmol g^−1^ h^−1^). The selectivity for CO was found to be 85%. The CdS/UiO-bpy/Co composites (Co loading 6.18 wt%) enhanced the separation and migration of the photo-induced charge carriers, thereby enhancing the CO_2_ adsorption for better photocatalytic performance. The CO_2_ reduction activity was studied using the photocatalyst in a mixer solution of acetonitrile and triethanolamine under visible light irradiation, where triethanolamine (TEOA) was used as an electron donor and acetonitrile as the solvent. The CO generation was increased with irradiation time. The CO evolution rate for CdS with Co-bpy as the co-catalyst was 110 μmol g^−1^ h^−1^, which was lower as compared to the CdS/UiO-bpy/Co composites. Here, the CdS nanoparticle size was found to be smaller due to the presence of the bpydc bridging ligands.

**Fig. 5:**
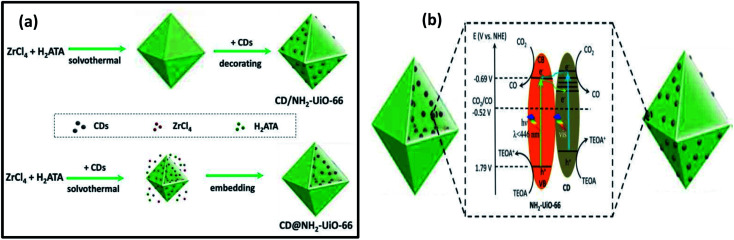
(a) Schematic illustration of two synthetic routes toward the CD-decorated NH_2_-UiO-66 particle and CD-embedded NH_2_-UiO-66 particle, respectively. The decorating strategy is based on different surface charges, and the encapsulating strategy is based on using CDs as seeds. (b) Electron transfer process and photocatalytic mechanism of two samples for CO_2_ reduction. These figures have been adapted from ref. 46 with permission from the ROYAL SOCIETY OF CHEMISTRY, copyright 2020.

Considering the potential use of carbon dots (CDs) that can act as both electron receptors and photosensitizers, Li *et al.* reported on CD-containing MOF photocatalysts for improving the CO_2_ reduction activity.^46^ They synthesized two types of photocatalysts for this study, one was CD decorated and the other one was the CD-embedded NH_2_-UiO-66 particles. During the study, they found that the positioning of CDs (28 mg mL^−1^) in MOFs greatly affects the photocatalytic activity of NH_2_-UiO-66 where CDs act as co-catalysts. The embedded CDs in NH_2_-UiO-66 exhibited better activity for CO_2_ reduction than the CD-decorated NH_2_-UiO-66. From the charge kinetic investigations, they confirmed that the embedded CDs were more suitable for creating charge separation and promoting charge transfer in MOFs than the decorated CDs in MOFs. This is due to the possibility of a direct contact of the embedded CDs with the internal Zr–O clusters of MOFs, which builds up many small heterojunctions. In this work, CDs not only act as electron receptors for charge separation, but also act as photosensitizers in the photocatalyst, which enhance its photocatalytic activity for CO_2_ reduction. They also confirmed that excessive CDs into MOFs can do harm to the photocatalytic activity. The photocatalytic activity was carried out using a Xe lamp (set at 100 mW cm^−2^) as the light source with a UV cut-off filter to achieve visible light at >420 nm. The catalyst was dispersed in a CO_2_ saturated solution of CH_3_CN and triethanolamine, where TEOA was used as the sacrificial agent. The CO production rate was 3.5 μmol gcat^−1^ h^−1^ with the pristine NH_2_-UiO-66 catalyst. For the CDs decorated MOF (CD/NH_2_-UiO-66), the rate was slightly increased to 4.0 μmol gcat^−1^ h^−1^. The CO production for CDs embedded MOF (CD@NH_2_-UiO-66) was quite high at 16.6 μmol gcat^−1^ h^−1^, which was 4.7 times greater than the pristine NH_2_-UiO-66 MOF. This confirms the importance of the ‘location effect’ of CDs in pristine MOFs for enhanced photocatalytic CO_2_ conversion application (Fig. 5).

Maina *et al.* reported on one zeolitic imidazolate framework (ZIF-8) with controlled encapsulation of TiO_2_ and Cu-TiO_2_ nanoparticles using the rapid thermal deposition (RTD) method.^47^ The photocatalytic performance of this composite towards CO_2_ conversion was studied in the presence of UV irradiation for 6 h reaction cycle. The CO_2_ conversion using a hybrid membrane reactor yielded methanol (CH_3_OH) and carbon monoxide (CO), and the yield was strongly dependent on the amount and configuration of the dopant semiconductor particles. The CuTiO_2_ nanoparticles (7 μg)-doped ZIF-8 membranes exhibited the highest photocatalytic performance of the CO production yield, and was enhanced by 233% compared to the pristine ZIF-8 membrane. Furthermore, the methanol yield was enhanced by 70%. This work provides a new path to fabricate hybrid membranes encapsulating inorganic semiconducting nanoparticles and MOFs, with enhanced application in various fields. The photocatalytic efficiency of CO_2_ conversion using hybrid membrane reactors was monitored taking both dimethylacetamide (DMAc) and acetonitrile (MeCN) as solvents. However, the photocatalyst exhibited the highest CO_2_ conversion performance for methanol and carbon monoxide production using DMAc as a solvent as compared to MeCN. This is due to the high CO_2_ solubilization capacity and stability of DMAc under UV light source. Furthermore, due to the higher Lewis's basicity of DMAc (0.73 B_KT_) than MeCN (0.23 B_KT_), DMAc exhibits more close interaction with CO_2_ (which is a Lewis acid), thereby exhibiting favorable photo-conversion.

**Fig. 6:**
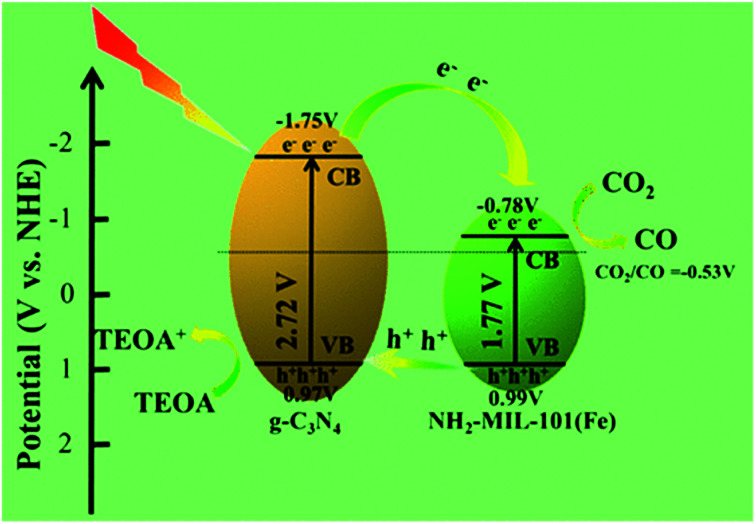
Possible route for the photoreduction of CO_2_ to CO under visible light using MCN-X heterostructures. This figure has been adapted from ref. 48 with permission from the AMERICAN CHEMICAL SOCIETY, copyright 2020.

Sun *et al.* reported on the heterostructures of NH_2_-MIL-101(Fe)/g-C_3_N_4_ (MCN-X) as photocatalysts with tunable surface structures, electrochemical, optical, and physicochemical properties to improve the photocatalytic CO_2_ reduction rate *via* a solvent-free route.^48^ Among these heterogeneous photocatalysts, NH_2_-MIL-101(Fe)/g-C_3_N_4_-30 wt% (MCN-3) exhibited an excellent CO_2_ to CO conversion rate of 132.8 μmol g^−1^, which was 3.6 times more than that of pristine NH_2_-MIL-101(Fe) and 6.9 times larger than that of sole g-C_3_N_4_. The NH_2_-MIL-101(Fe)/g-C_3_N_4_ composite with efficient interface electron transfer between NH_2_-MIL-101(Fe) (Fe ∼74%) and g-C_3_N_4_ resulted in the improved photocatalytic CO_2_ reduction upon visible light irradiation. This visible light-driven photoreduction of CO_2_ by the MCN-X series was performed *via* a solvent-free route by loading the photocatalysts in the filter membrane, and employing TEOA as the sacrificial agent. During the visible light driven photoreduction process, the heterostructure photocatalyst exhibited no HCOOH, CH_3_OH, H_2_ and CH_4_ generation as products due to the employed solvent-free process, which was confirmed by ^1^H NMR spectral measurement and GC measurement. The only product formation was CO by MCN-3 with a higher photocatalytic efficiency rate of 132.8 μmol g^−1^ as compared to individual g-C_3_N_4_ and NH_2_-MIL-101(Fe). MCN-4 with an increased amount of g-C_3_N_4_ compared to MCN-4 reduced the catalytic activity, which may be attributed to the excess g-C_3_N_4_ over the NH_2_-MIL-101(Fe) surface that blocks the interaction between CO_2_ and the catalytic active sites. The proves the presence of a synergistic effect between the NH_2_-MIL-101(Fe) and g-C_3_N_4_. The GC-MS analysis ensures the formation of CO during the photoreduction of CO_2_ (Fig. 6).

Chen and Duan *et al.* reported on the Au/PPF-3 composite, hybrids of thin porphyrin paddle-wheel frameworks-3 (PPF-3) nanosheets (PPF-3_1) anchored with AuNPs for the photocatalytic CO_2_ conversion into HCOOH.^49^ This helped to improve the morphology and assembly mode of PPF-3 between the AuNPs and PPF-3 nanosheets. Thin nanosheets in the photocatalytic process cause a quick charge transfer and high mass transport compared to thick nanosheets. Alternatively, nanosheets loaded with AuNPs will result in more effective light absorption than the AuNPs encapsulated nanosheets. The Au/PPF-3 composite exhibits better CO_2_ conversion in the MeCN/EtOH system by plasmon resonance energy transfer process than pure PPF-3_1 or hybrids of thick PPF-3 nanosheets (PPF-3_2) supported AuNPs. PPF-3 nanosheets were accumulated with plasmonic AuNPs by electrostatic interaction to further enhance the light absorption ability of the Au/PPF-3 hybrids. The photocatalytic HCOOH production rate using Au/PPF-3_1A was ∼4-fold higher than sole pristine PPF-3_1 in the presence of visible light. The aspect ratio of the Au nanoparticles and PPF-3 MOF were 1 : 10 with 16% Co content in PPF-3 MOF for good CO_2_ conversion activity. Through this work, they provided a potential and novel way to improve the rate of the photocatalytic activity by improving the MOFs framework *via* morphological assembly route.

**Fig. 7:**
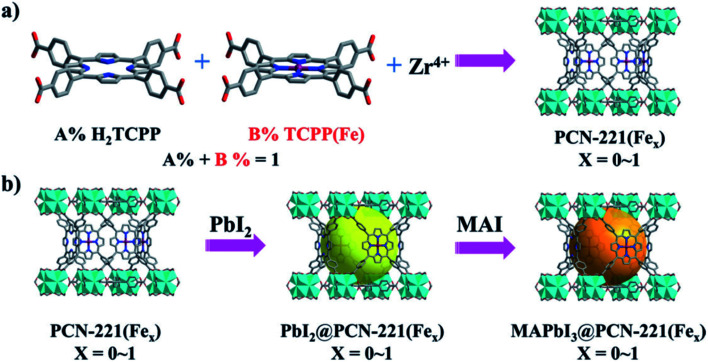
Schematic illustrations for the synthesis of (a) PCN-221(Fex), and (b) MAPbI_3_ QDs (large spheres) encapsulated in the pores of PCN-221(Fex) by a sequential deposition route (MAI = CH_3_NH_3_I). This figure has been adapted from ref. 50 with permission from WILEY-VCH, copyright 2019.

Zhang *et al.* reported one Fe-porphyrin based-MOF, MAPbI_3_@PCN-221(Fe_0.2_) encapsulating the Perovskite quantum dots, CH_3_NH_3_PbI_3_ (MAPbI_3_) for CO_2_ photoreduction into CO and CH_4_.^50^ The perovskite QDs in the MOF transfer the photogenerated electrons of QDs to the Fe catalytic site in the MOF to enhance the photocatalytic activity with 40% and 12% Zr and Fe contents in MOF, respectively. Using water as the source of an electron (sacrificial reductant), the MAPbI_3_@PCN-221(Fe_0.2_) photocatalyst exhibits a high photocatalytic CO_2_ reduction yield of 1559 μmol g^−1^ to CO (34%) and CH_4_ (66%), which is 38-fold larger than PCN-221(Fe_0.2_) in the absence of perovskite QDs. Moreover, the MAPbI_3_ QDs integration into MOF can significantly enhance the stability of both LHP QDs and PCN-221(Fex). The photocatalytic CO_2_ reduction experiments were performed in a CO_2_-saturated ethyl acetate solution containing a little water as the sacrificial reductant and the solution was irradiated using 300 W Xe-lamp with a 400 nm filter, which yielded CO and CH_4_ as the main reaction products for all PCN-221(Fex) and MAPbI_3_@PCN-221(Fex) photocatalysts containing Fe. In the absence of Fe in the system, CO was found as the only product for PCN-221 and MAPbI_3_@PCN-221 with little photocatalytic activity. For the photocatalytic systems, no H_2_ and other liquid products like CH_3_OH and HCOOH were detected under similar irradiation time of 25 h. The presence of Fe plays a vital role for photocatalytic CO_2_ reduction. MAPbI_3_@PCN-221(Fe_0.2_) exhibited the highest yield for CO_2_ conversion, leading to CO (104 μmol g^−1^) and CH_4_ (325 μmol g^−1^) (Fig. 7).

Li and his co-workers reported on the UiO-66-NH_2_/graphene composite synthesized by *in situ* assembly of UiO-66-NH_2_ onto graphene *via* microwave synthesis method for CO_2_ conversion into formic acid and methane.^51^ UiO-66-NH_2_ crystals were highly dispersed on the graphene surface, and junctions between the graphene surface and UiO-66-NH_2_ was formed. The UiO-66-NH_2_/2.0 graphene (2 wt% graphene) composite was capable of capturing CO_2_ ∼73 cm^3^ g^−1^ and exhibited high performance towards CO_2_ conversion into formic acid (35.5 μmol) with good selectivity (78.6%) under visible-light irradiation. The high photocatalytic activity for the photoreduction of CO_2_ with high selectivity was attributed to the small crystals size of MOFs, strong CO_2_ sorption, well-dispersion of MOF on graphene, and strong junction between MOF and graphene. This leads to the generation of more charge carriers by trapping light and quick electron transfer from bulk to the MOF surface, increasing the stability of the system. The presence of amino groups in the MOFs structure enhances the CO_2_ adsorption capability due to their basic nature. The introduction of graphene reduced the competitive reaction of hydrogen evolution (13.2 μmol) for the UiO-66-NH_2_/2.0 graphene composite as compared to pure UiO-66-NH_2_ (16.9 μmol), and it led to efficient formic acid production during photocatalytic CO_2_ conversion. Introducing graphene made the composite highly selective towards formic acid production compared to hydrogen evolution during the CO_2_ photoreduction. The high selectivity of CO_2_ reduction towards formic acid formation as compared to hydrogen evolution using the UiO-66-NH_2_/graphene composite can be attributed to the higher CO_2_ adsorption and highly negative conduction band (CB) potential of the composite (−0.82 eV) that stimulates the higher reduction of CO_2_ into HCOOH. The photocatalytic CO_2_ reduction activity was carried out using 300 W Xe lamp, *λ* >410 nm as light source (irradiation time ∼4 h), and DMF was used as solvent. TEOA was used as a sacrificial agent and H_2_O (3 mL) was used as a proton source for the photoreduction of CO_2_ (Table 1).

**Table 1:** MOFs composites for photocatalytic CO_2_ conversion

Sl no.	MOF composite or photocatalyst	Light source	Solvent used	Sacrificial agent	Fuel product	Photocatalytic reaction rate	Ref.
1	[Re^I^(CO)_3_(dcbpy)Cl]-doped UiO-67	450 W Xe lamp with 300 nm cut-off filter	MeCN : triethylamine = 20 : 1	TEA	CO	CO-TON: 5	36
2	PCN-222	Xe lamp, wavelength ∼420–800 nm	MeCN/TEOA (10 : 1 v/v, 60 mL)	TEOA	HCOO^−^	30 μmol g^−1^ h^−1^	37
3	Ag ⸦ Re_3_-MOF	300 W Xe lamp with visible band pass filters, *λ* = 400–700 nm	Acetonitrile/triethylamine	TEA	CO	TON: 2.8	39
4	CdS/UiO-bpy/Co composite	300 W Xe lamp, *λ* ≥420 nm	Acetonitrile/triethanolamine	TEOA	CO	235 μmol g^−1^ h^−1^	45
5	CDs embedded MOF (CD@NH_2_-UiO-66)	Xe lamp (set at 100 mW cm^−2^) UV-cut-off filter∼ *λ* >420 nm	CH_3_CN/triethanolamine	TEOA	CO	16.6 μmol gcat^−1^ h^−1^	46
6	CuTiO_2_ doped ZIF-8	UV-lamp, *λ* = 320–480 nm	Dimethylacetamide (DMAc)/acetonitrile (MeCN)	TEOA	CO, CH_3_OH	CO ∼2170 ppm g^−1^ cat^−1^	47
						CH_3_OH ∼2238 ppm g^−1^ cat^−1^	
7	NH_2_-MIL-101(Fe)/g-C_3_N_4_ (MCN-3)	Visible light, 6 h irradiation	Solvent free route	TEOA	CO	132.8 μmol g^−1^	48
8	Au-PPF-3	Visible light	MeCN/EtOH	EtOH	HCOOH	42.7 μmol g^−1^ h^−1^	49
9	MAPbI_3_@PCN-221(Fe)	300 W xenon lamp	EtOAc/H_2_O	H_2_O	CO, CH_4_	6.6 μmol g^−1^ h^−1^ (CO)	50
						12.9 μmol g^−1^ h^−1^ (CH_4_)	
10	UiO-66-NH_2_/2.0graphene	*λ* >410 nm, 4 h irradiation	DMF/TEOA/H_2_O	TEOA	HCOO^−^, H_2_, CH_4_	35.5 μmol (HCOO^−^)	51
						13.2 μmol (H_2_)	
						0.90 μmol (CH_4_)	

#### (b) Electrocatalytic approach

Electrocatalytic CO_2_ reduction is another important approach for the production of valuable chemicals and fuels. This approach is very helpful for CO_2_ reduction considering the high reaction rates under ambient conditions, easy tuning of the overpotential value and voltage-dependent products. Still, challenges lie in the architecture of the catalysts, which can produce good catalytic efficiency with low applied potential, high faradaic efficiency, long-term stability of the reaction, good solubility of CO_2_ in the electrolyte solution, low temperature working condition, and the use of a nonaqueous electrolyte solution. The faradaic efficiency is an important key for the electrochemical CO_2_ conversion into value-added fuels and chemicals. Some catalysts exhibit excellent efficiency towards selective product formation in CO_2_ conversion. Catalysts executing CO_2_ conversion with low overpotential, high current density and high faradaic efficiency values are considered as the best catalysts for this purpose. Despite having several good reports for electrocatalytic CO_2_ conversion with good faradaic efficiency, scientists are facing various difficulties on developing excellent catalysts towards the selective product formation of CO_2_ reduction. This is due to the high overpotential value owing to the symmetrical structure of CO_2_ and higher oxidation state of carbon, low charge transfer kinetics or low current density, consistency of the electrodes for shorter periods, CO_2_ concentration, reaction temperature and pressure, pH, solvent selection, and product selectivity, which are the main reasons affecting the efficiency in the electrocatalytic CO_2_ reduction limiting their practical uses to some extent. MOFs-based composites are considered as an effective heterogenous catalyst for the electrocatalytic conversion of CO_2_ into value-added chemicals and fuels considering their unique properties. The heterogeneous electrochemical reduction of CO_2_ occurs at the electrode–electrolyte interfaces following these steps:^31^

(i) chemical adsorption of CO_2_ on the catalyst surface, where the cathodic electrode acts as a catalyst.

(ii) Electron or proton migration for C–O bond breaking and C–H bond formation.

(iii) Product species rearrangement, followed by desorption from the surface of the electrode and diffusion into the electrolyte.

Schroder *et al.* reported on one MOF-based electrode as an electrocatalyst prepared by electro-synthesis of MFM-300(In) on indium foil to study electrochemical CO_2_ reduction.^52^ Since the efficient CO_2_ electro-reduction over the MOFs materials is suppressed due to the poor interaction between the electrode surface and thermally synthesized MOFs, it results in a low faradaic efficiency of the particular product and low electrochemical stability of the catalyst. The synthesized electrocatalyst, MFM-300(In)-e/In electrode, exhibits improvement in the conductivity as compared to the MFM-300(In)/carbon-paper electrodes. The resultant electrocatalyst, MFM-300(In)-e/In, exhibited exceptional activities for the electro-reduction of CO_2_ with a current density of 46.1 mA cm^−2^ at an applied potential of −2.15 V *vs.* Ag/Ag^+^. The electrocatalytic CO_2_ reduction results in the formation of formic acid after 2 h electrocatalysis with an exceptional faradaic efficiency of 99.1%. Indium and [NiS_4_] sites at the linker are responsible for the CO_2_ absorption activity. The facile preparation of the MFM-300(In)-e/In electrode with excellent electrochemical stability affords a new pathway for the development of efficient electro-catalysts for CO_2_ reduction. They have also reported that the electro-synthesized MOF integrates additional framework In^3+^ sites as structural defects, which enhances the charge transfer capability and promotes the CO_2_ activation to the radicals, maintaining the stability and excellent electrocatalytic activity of the catalyst. The catalysis mechanism was studied by density functional theory (DFT).

Liu *et al.* reported a novel bismuth-based organic framework (Bi-BTC-D MOF) for the electrochemical reduction of CO_2_.^53^ The Bi-MOF obtained by the conventional hydrothermal synthesis method was found to be very stable and effective to ERCO_2_. The resultant Bi-BTC-D MOF (42% Bi in MOF) produced formate as a product with a Faraday efficiency (FE) of 95.5% at a potential of −0.86 V_RHE_ and a current density of −11.2 mA cm^−2^. The FE was found to be very effective even after 12 h of spontaneous electrolysis without significant reduction. The experimental analysis confirmed that the excellent catalytic performance was attributed to the morphology of the electrocatalyst. DFT results confirmed that the Bi sites of Bi-BTC-D MOF played a major role in the efficient HCOO^−^ production at very low overpotential value. The BTC and DMF ligands in the Bi-BTC-D structure control the catalytic activity of the Bi atoms effectively. This work reveals the potential uses of the MOFs materials for the electroreduction of CO_2_ into sustainable fuels.

**Fig. 8:**
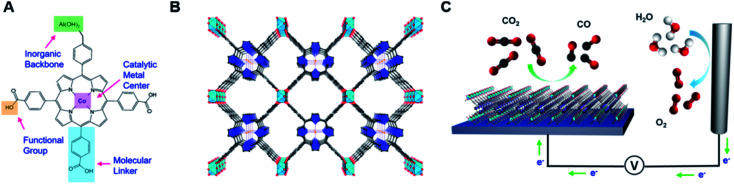
The MOF catalyst allows for the modulation of metal centers, molecular linkers, and functional groups at the molecular level (A). The organic building units, in the form of cobalt-metalated TCPP, are assembled into a 3D MOF, Al_2_(OH)_2_TCPP-Co with variable inorganic building blocks (B). Co, orange spheres; O, red spheres; C, black spheres; N, blue spheres; Al, light-blue octahedra; and pyrrole ring, blue. In this structure, each carboxylate from A is bound to the aluminum inorganic backbone. The MOF is integrated with a conductive substrate to achieve a functional CO_2_ electrochemical reduction system (C). This figure has been adapted from ref. 54 with permission from the AMERICAN CHEMICAL SOCIETY, copyright 2015.

Yaghi and Yang *et al.* reported on thin films of nanosized MOFs as nanoscopic materials for the efficient and selective reduction of CO_2_ to carbon monoxide in aqueous electrolytes.^54^ They synthesized cobalt porphyrin MOF, Al_2_(OH)_2_TCPP-Co (TCPP-H_2_ = 4,4′,4′′,4′′′-(porphyrin-5,10, 15, 20 tetrayl)tetrabenzoate) for CO production with 76% selectivity and stability up to 7 h with per-site turnover number (TON) of 1400. From *in situ* spectroelectrochemical analysis, they confirmed the cobalt oxidation state during the course of the reaction, where they found that the maximum catalytic centers in this MOF electrocatalyst are redox-accessible, where Co(ii) is reduced to Co(i) during catalysis. They further confirmed 6.1 × 10^16^ Co-atoms per square centimeter loading on MOF for the significant CO_2_ reduction to CO. This study signifies the development of MOF-based electrochemical CO_2_ reduction methods, where the catalytic active site, thickness/loading and inorganic backbone were chosen rationally, and the resulting MOF was incorporated into a conductive backbone. Such type of system modularity results in different prospects to further enhance the performance and develop new ways in electrocatalysis (Fig. 8).

Albo and his co-workers reported on Cu-based metal–organic porous materials (MOPM) supported on gas diffusion electrodes (GDEs) to accelerate the electrocatalytic CO_2_ conversion to alcohols.^55^ The four different synthesized MOPM-GDEs were named as, (1) HKUST-1 MOF, [Cu_3_(μ_6_-C_9_H_3_O_6_)_2_]_*n*_; (2) CuAdeAce MOF, [Cu_3_(μ_3_-C_5_H_4_N_5_)_2_]_*n*_; (3) CuDTA mesoporous metal–organic aerogel (MOA), [Cu(μ-C_2_H_2_N_2_S_2_)]_*n*_; (4) CuZnDTA MOA, [Cu_0.6_Zn_0.4_(μ-C_2_H_2_N_2_S_2_)]_*n*_. Theses electrodes exhibited comparatively large surface areas, large accessibility to the Cu catalytic centers, and favorable electrocatalytic CO_2_ reduction activity with efficient production of methanol and ethanol in the liquid phase. The electrocatalytic reduction activity was carried out in a filter-press electrochemical cell under ambient conditions. The faradaic efficiencies of the electrodes for CO_2_ conversion were obtained as HKUST-1 (15.9%), CuAdeAce (1.2%), and CuZnDTA (9.9%) at current density of 10 mA cm^−2^. Among the four electrodes, the HKUST-1- and CuZnDTA-based electrodes exhibited stability in the electrocatalytic performance for up to 17 and 12 h, respectively. This work provides an idea to design efficient electrocatalysts for CO_2_ reduction, including paddlewheel motifs that exhibit square planar coordination geometry around the Cu(ii) centers and create open metal sites to cause strong interaction with guest species over the porous framework.

Dong and Feng *et al.* reported on one effective bimetallic two-dimensional conjugated metal–organic framework (2D c-MOF) named as PcCu-O_8_-Zn with copper-phthalocyanine (CuN_4_) as ligand and zinc-bis (dihydroxy) complex (ZnO_4_) as a linkage for the electrocatalytic CO_2_ reduction reaction (CO_2_RR).^56^ The PcCu-O_8_-Zn exhibited high selectivity towards CO (88%) with simultaneous water reduction to H_2_ (12%). The TOF exhibited by PcCu-O_8_-Zn was 0.39 s^−1^ with long-term stability (>10 h). Varying the metal centers and the applied potential, the molar ratio of H_2_/CO can be tuned, thereby upgrading the use of 2D c-MOFs for syngas industry applications. The spectroelectrochemistry and theoretical calculation reveals the synergistic catalytic mechanism of the work, where ZnO_4_ complexes act as catalytic sites for CO_2_RR, while CuN_4_ centers cause the protonation of adsorbed CO_2_ during the CO_2_ reduction process. This work provides an idea to develop bimetallic MOFs electrocatalysts for syngas synthesis by synergistically catalyzing CO_2_RR.

**Fig. 9:**
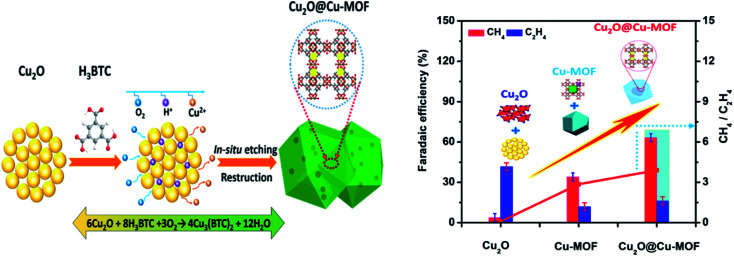
Schematic illustration of the process to synthesize Cu_2_O@Cu-MOF and the bar diagram showing the faradaic efficiency of generated hydrocarbons using the synthesized catalysts. This figure has been adapted from ref. 57 with permission from the AMERICAN CHEMICAL SOCIETY, copyright 2019.

Yu and Qiu *et al.* reported on one tailormade multifunctional Cu-MOF as an electrocatalyst, which was synthesized from Cu_2_O to Cu_2_O@Cu-MOF by time-resolved controllable restructuration.^57^ The synthesized Cu_2_O@Cu-MOF electrocatalyst (Cu ∼33%) showed a time-responsive nature and large specific surface area for the strong chemisorption of CO_2_ on the catalytically active sites of the Cu_2_O surface of the MOF composite. The generated charge-transfer was derived from the Cu_2_O core, rather than the Cu-MOF. The Cu_2_O@Cu-MOF electrocatalyst yielded hydrocarbons with a high hydrocarbon faradaic efficiency of 79.4%. The faradaic efficiency of CH_4_ was found to be quite high at 63.2% at −1.71 V *vs.* RHE. The multiple functionalities (like catalysis, absorption capability and activation) of the MOF obtained from the combined effect of Cu_2_O and Cu-MOF are responsible for the high electrocatalytic performance and selective reduction of CO_2_ to CH_4_ (Fig. 9).

Heterogeneous electrocatalysts have the ability to ease the deactivation (like aggregation and dimerization) of active catalysts. Thus, these catalysts can improve the catalytic lifetime, catalyst's solubility in any solvent, and controllability over surroundings of the catalyst's active sites for enhanced performance. Heterogeneous catalysts have the capability to keep away the reduction catalyst from the electrode, in which the oxidation half-reaction (*e.g.*, water to O_2_) occurs. Based on the principle of heterogeneous electrochemical conversion of CO_2_ to fuels under the requirement of high flux conditions to aggregate large reactant accessible catalysts on conductive surfaces *via* molecular catalysis, Hod and his group reported on the electrophoretic deposition of thin films of a perfectly chosen MOF material for electrocatalytic CO_2_ reduction into useful chemicals.^58^ This was an effective method for immobilizing the required catalyst quantity. They reported on one Fe-porphyrin incorporated MOF, where the functionalized Fe-porphyrins behaved as structurally and catalytically competent, and as redox-conductive linkers. This approach resulted in the highly effective surface coverage of electrochemically addressable catalytic sites (∼10^15^ sites per cm^2^). The resulting MOF composite produced CO and H_2_ as CO_2_ reduction products with ∼100% faradaic efficiency. The well-defined porosity of the MOF enabled the reactant, solvent and electrolyte access to the surface of the catalytic sites. The metalloporphyrin in MOF helps in the delivery of reducing equivalents to catalytic sites, which are not in direct contact with the underlying electrode.

**Fig. 10:**
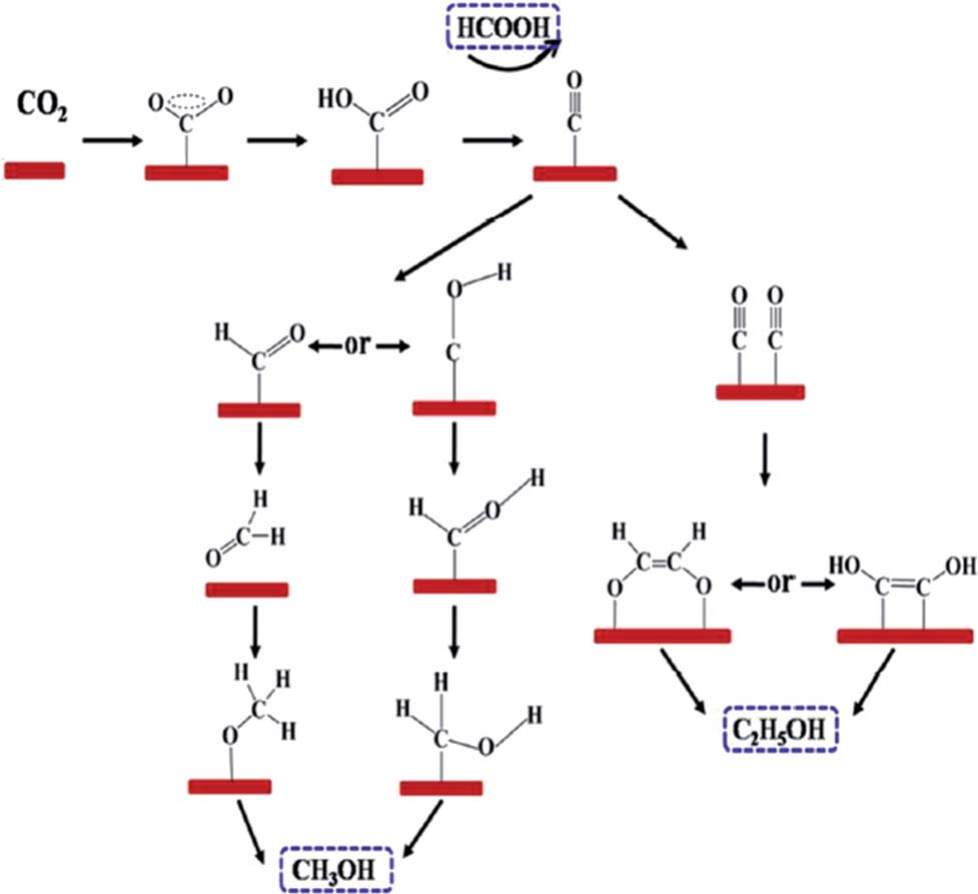
Proposed reaction paths for CO_2_ electroreduction on OD Cu/C-1000, producing formic acid (HCOOH), methanol (CH_3_OH), and ethanol (C_2_H_5_OH). This figure has been adapted from ref. 59 with permission from the AMERICAN CHEMICAL SOCIETY, copyright 2017.

Quan *et al.* reported on oxide-derived Cu/carbon (OD Cu/C) catalysts by a facile carbonization of Cu-based MOF (HKUST-1).^59^ The electrocatalyst exhibited good selectivity, good stability and high activity towards electrochemical CO_2_ reduction to alcohol products. The resultant catalyst, OD Cu/C-1000, yielded a faradaic efficiency of 13.8–8.3% and 31.4–34.8% for methanol and ethanol at −0.5 to −0.7 V, respectively *vs.* RHE. The onset potential for C_2_H_5_OH production was very low, which was approx. −0.1 V (*vs.* RHE), corresponding to an overpotential value of 190 mV. The excellent enhancement in the selectivity and activity of the electrocatalyst was attributed to the synergistic effect between the highly dispersed copper and matrix of porous carbon. For a mechanistic study for the electrocatalytic CO_2_ reduction on OD Cu/C electrode, *in situ* infrared spectrum was employed. Based on DFT, they proposed the possible reaction pathway for CO_2_ electroreduction on the OD Cu/C-1000 electrode, as shown in Fig. 10 (Table 2).

**Table 2:** MOFs composites for the electrocatalytic CO_2_ conversion

Sl no.	Electrode	Electrolyte	Potential	Product	Faradaic efficiency (%)	Ref.
1	MFM-300(In)-e/In	0.5 M Emim BF_4_/MeCN	−2.15 V *vs.* Ag/Ag^+^	HCOOH	99.1	52
2	Bi-BTC-D MOF	0.5 M KHCO_3_ aqueous solution	−0.86 V_RHE_	HCOO^−^	95.5	53
3	Cobalt-porphyrin MOF	0.5 M K_2_CO_3_	−0.5 to −0.9 *vs.* RHE	CO	76	54
4	Cu-based MOPM-GDEs	0.2 M KHCO_3_	−0.9 to −1.75 V *vs.* Ag/AgCl	CH_3_OH, C_2_H_5_OH	(CH_3_OH + C_2_H_5_OH) ∼HKUST-1 (15.9), CuAdeAce (1.2), CuZnDTA (9.9)	55
5	PcCu-O_8_-Zn	0.1 M KHCO_3_ aqueous solution	−0.7 V *vs.* RHE	CO, H_2_	CO ∼88	56
					H_2_ ∼12	
6	Cu_2_O@Cu-MOF	0.1 mol L^−1^ KHCO_3_	−1.71 V *vs.* RHE	CH_4_, C_2_H_4_	CH_4_ ∼63.2	57
					C_2_H_4_ ∼16.2	
7	Fe-porphyrin incorporated MOF (Fe_MOF-525)	1 M TBAPF_6_ acetonitrile	−1.3 V *vs.* NHE	CO, H_2_	(CO + H_2_) ∼100	58
8	OD Cu/C	0.1 M KHCO_3_	−0.5–0.7 V *vs.* RHE	CH_3_OH, C_2_H_5_OH	CH_3_OH (3.8–8.3), C_2_H_5_OH (31.4–34.8)	59

#### (c) Photoelectrocatalytic approach

Photoelectrochemical (PEC) CO_2_ reduction into fuels and useful chemicals is one of the most promising approaches. Yet, there are many challenges as few reports are found using MOF-based materials for photoelectrocatalytic CO_2_ conversion. The PEC approach is an artificial photosynthesis method to produce valuable hydrocarbons that mainly uses the p-type semiconductor as a photocathode to conduct CO_2_ reduction. The PEC of CO_2_ follows both photocatalytic and electrocatalytic strategies. Thus, it utilizes the light energy sources to reduce the electricity consumption as compared to the electrocatalytic approach. The PEC approach results in an efficient catalytic rate due to the application of an external bias voltage that helps to promote the separation of photogenerated charge-carriers, which is very important to achieve high catalytic efficiency. No complete studies on PEC CO_2_ conversion mechanism have been made until now. One H-type PEC reactor has been introduced with a suitable ion-exchange process in most of the cases. Also, PEC reactor configurations (like microfluidic, continuous flow, and PV) have attained great importance in recent years for their high performance in this regard.^60^

**Fig. 11:**
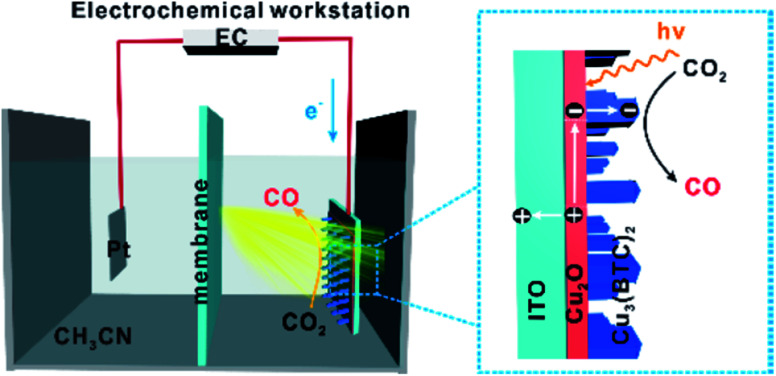
Schematic illustration for PEC CO_2_ reduction in acetonitrile using a hybrid Cu-MOF/Cu_2_O photocathode (unscaled). This figure has been adapted from ref. 61 with permission from the AMERICAN CHEMICAL SOCIETY, copyright 2019.

The p-type semiconductors such as Cu_2_O exhibit harmful photo-corrosion and chemical changes. In view of this, Xiong *et al.*^61^ reported on the Cu_3_(BTC)_2_/Cu_2_O photocathode by coating Cu_3_(BTC)_2_ MOF on the Cu_2_O photocathode, which has the ability to stop the photo-corrosion of Cu_2_O and develop active catalytic sites for CO_2_ reduction. By ultrafast spectroscopy, it was found that the newly formed interface can effectively enhance the charge separation and charge transfer. The MOF coating can enhance the activity and durability of Cu_2_O for PEC CO_2_ reduction. This work gives insight into the architecture of progressive hybrid photoelectrodes and the importance of interfacial charge dynamics in photoelectrocatalytic CO_2_ conversion. The Cu-MOF/Cu_2_O photocathode converted CO_2_ to CO with a faradaic efficiency of 95% without using any sacrificial agent, as shown in Fig. 11.

In this work, photoelectrochemical (PEC) experiments were performed using a three electrode quartz photoelectrochemical cell under irradiation, combined with an electrochemical workstation and a gas chromatograph (GC). The analysis was done using CO_2_-saturated acetonitrile containing 0.1 M tetrabutylammonium hexafluorophosphate as a supporting electrolyte, and a 300 W xenon lamp with a 420 nm long wave-pass cut-off filter (*λ* >420 nm) was used as the light source. The applied external potential for CO production was about −1.77 and −1.97 V *vs.* Fc/Fc^+^, which leads to 95% FEs (Table 3).

**Table 3:** Presenting some MOFs towards the selective product formation during CO_2_ conversion

Catalyst	Light source & time	Solvent	Product	Yield (μmol g^−1^) & selectivity (%)	Ref.
CsPbBr_3_ QDs (15%)/UiO-66(NH_2_)	300 W xenon arc lamp (≥420 nm), 12 h	H_2_O/ethyl acetate	CO, CH_4_	98.57, 3.08	62
NH_2_-rGO (5 wt%)/Al-PMOF	125 W mercury lamp, 6 h	MeCN/TEOA	HCOO^−^	4113.6 (100%)	63
NH_2_-MIL-101(Fe)	300 W xenon arc lamp (400 < *λ* < 780 nm), 5 h	Solvent free	CO	87.6	64
Eu–Ru(phen)_3_-MOF	300 W (420 < *λ* < 800 nm), 6 h	MeCN/TEOA	HCOO^−^	2205	65
NH_2_-UiO-66(Zr)	500 W (420 < *λ* < 800 nm), 6 h	MeCN/TEOA	HCOO^−^	2198 (72%)	66
Copper porphyrin MOF	300 W Xe arc lamp (*λ* ≥ 420 nm), 1 h	H_2_O/TEA	CH_3_OH	262.6 (95%)	67
Ui-66-CrCAT	Visible, 1 h	MeCN/TEOA (4 : 1), 0.1 M BNAH	HCOOH	1724, 959	68
Ui-66-GaCAT					
PCN-22	Visible, 10 h	MeCN/TEOA	HCOO^−^	30 (51%)	69
Co-ZIF-9/TiO_2_	UV-vis, 10 h	H_2_O	CO	8.8 (31%), 0.99 (3%), 1.30 (5%)	70
			CH_4_		
			H_2_		
PCN-136	300 W xenon arc lamp (≥420 nm), TIPA, 12 h	MeCN/H_2_O	HCOO^−^	10.52 (17%)	71
Co_6_-MOF	150 W xenon lamp (420 ≤ *λ* ≤ 780), TEOA, 3 h	MeCN/H_2_O	CO, H_2_	39.36 (71%), 28.13 (51%)	72
MOF-74	500 W xenon lamp, 5 h	H_2_O	CO	7.42	73
Pt/MOF-74			CO,CH_4_	8.85 (99.7%), 9.04	
Au@Pd@MOF-74			CO	12.31 (100%)	
Pt/Au@Pd@MOF-74			CO, CH_4_	2.32 (99.6%), 12.35	
MOF-525	300 W xenon arc lamp (400 nm < *λ* < 800 nm), TEOA, 6 h	MeCN/TEOA	CO, CH_4_	384.12, 37.2	74
MOF-525-Co			CO, CH_4_	1203.6, 220.56	
MOF-525-Zn			CO, CH_4_	670.2, 69.81	
AUBM-4	150 W (420 < *λ* < 800 nm), TEOA, 6 h	MeCN/TEOA	HCOO^−^	2196	75
Cu_3_(BTC)_2_@TiO_2_	300 W xenon arc lamp, *λ* < 400 nm, 4 h	H_2_O	CH_4_, H_2_	2.64, 2.29	76
Ag@Co-ZIF-9	Visible light, 0.5 h	MeCN/TEOA/H_2_O	CO, H_2_	28.4, 22.9	77
g-CNQDs@MOF	Visible light, 24 h	DMF : H_2_O (3 : 1), TEA	CH_3_OH	9264	78 (our work)

### 2.2. Hydrogen production

Hydrogen is a clean energy carrier that has various useful applications as fuel in power generation, vehicles and industrial applications, such as manufacturing of nitrogenous fertilizers and many useful organic products. Hydrogen is considered as one of the most promising fuels to overcome energy limitations for future generation. Therefore, the efficient production of hydrogen chemically using low-cost, feasible reaction processes has become very challenging for boosting the hydrogen economy. Nowadays, the water splitting process is considered an efficient route for hydrogen production efficiently using solar energy and other energy sources, along with hydrogen generation by CO_2_ conversion process as discussed above. The design of efficient catalysts for this purpose is another important factor. Recently, many MOFs-based catalysts have been developed for hydrogen production considering the unique structural features of MOFs.^79^ Enhanced light absorption ability of MOFs accelerates the charge separation of photogenerated electron–hole pairs for efficient hydrogen evolution reactions. Recently, chemists have reported on many MOFs composites containing nanoparticles, g-C_3_N_4_, traditional inorganic semiconducting materials, complexes, COFs, metals, and other functional materials with enhanced H_2_ production rate.^80–86^ Many MOFs derivatives using MOFs as templates like oxides and sulfides have been reported for excellent H_2_ production.^87^

Water splitting reactions can proceed *via* three main routes: (a) photocatalytic water splitting, (b) electrocatalytic water splitting, and (c) photoelectrocatalytic water splitting. All three approaches are very convenient to carry out water splitting reactions for efficient hydrogen production. Recently, the photoelectrocatalytic approach has received much attention of the scientists due to the recent development, and considering that there are very few reports using both photo- and electro-catalytic methods.

#### (a) Photocatalytic approach

**Fig. 12:**
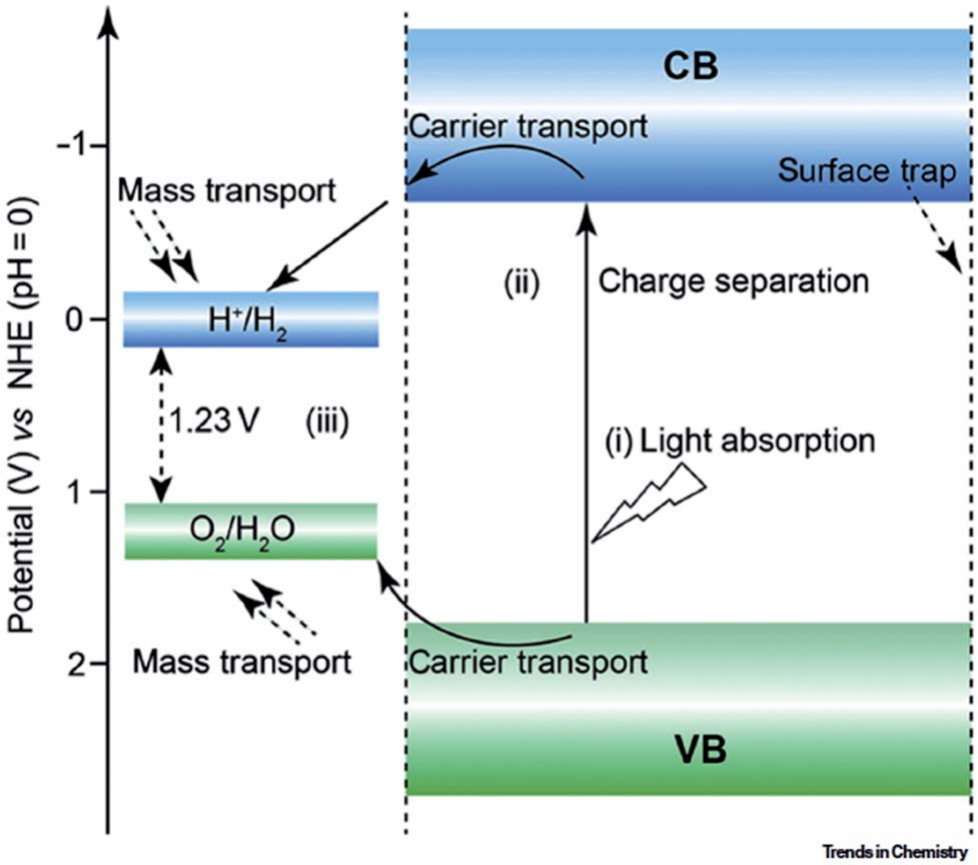
Basic mechanism for the photocatalytic hydrogen production. This figure has been adapted from ref. 90 with permission from CELL PRESS, copyright 2020.

Photocatalytic hydrogen production is a very promising approach, which can utilize solar energy for conversion into chemical energy. Water plays the important role for photocatalytic hydrogen production, owing to its important properties like most abundant resource on earth and pollution-free hydrogen generation with high calorific value of hydrogen. Moreover, photocatalytic water reduction leads to two half reactions, oxidation and reduction, where oxygen and hydrogen are produced on the surface of the photocatalyst, respectively.^88^ Water splitting is a 4 e^−^ process thermodynamically that requires a Gibbs free energy (Δ*G*) of 237 kJ mol^−1^, and it corresponds to a potential value of 1.23 eV.^88,89^ Therefore, the water splitting reaction uses more than 50% of solar energy, and the two half reactions acquire high energy to proceed. Hence, both thermodynamic and kinetic considerations are important for water splitting reactions. The photocatalytic H_2_-evolution efficiency mainly depends upon the nature and amount of the photocatalysts and the irradiation source. The photocatalytic water splitting follows three main steps: (a) irradiation of the semiconductor photocatalyst to generate electrons in the excited state of the photocatalysts (CB or LUMO), and holes are generated in the valence band (VB) or HOMO of the photocatalyst. (b) The photogenerated charge carriers get separated by migration to the catalyst's surface. (c) The excited electrons on the photocatalyst surface *i.e.*, in LUMO of the photocatalyst react with water to produce hydrogen, and oxygen is produced in the holes of the photocatalyst (HOMO). Desorption of gases from the surface of the photocatalyst then occurs. For a feasible water splitting reaction, the LUMO or CB of the photocatalyst must be more negative than the redox potential of water reduction (0.00 V *vs.* NHE, pH = 0), and the VB or HOMO of the photocatalyst must possess more positive potential as compared to the redox potential for water oxidation (1.23 V *vs.* NHE, pH = 0) (Fig. 12).

From the literature,^90^ it was found that photocatalytic water splitting (PWS) has three main approaches:

(i) photocatalytic overall water splitting (POWS) [2H_2_O → 2H_2_ + O_2_].

(ii) Photocatalytic partial water splitting (PPWS) [2H_2_O + sacrificial reagent → 2H_2_ + oxidized sacrificial reagent products].

(iii) Photocatalytic intermediate water splitting (PIWS) [2H_2_O → H_2_ + H_2_O_2_].

**Fig. 13:**
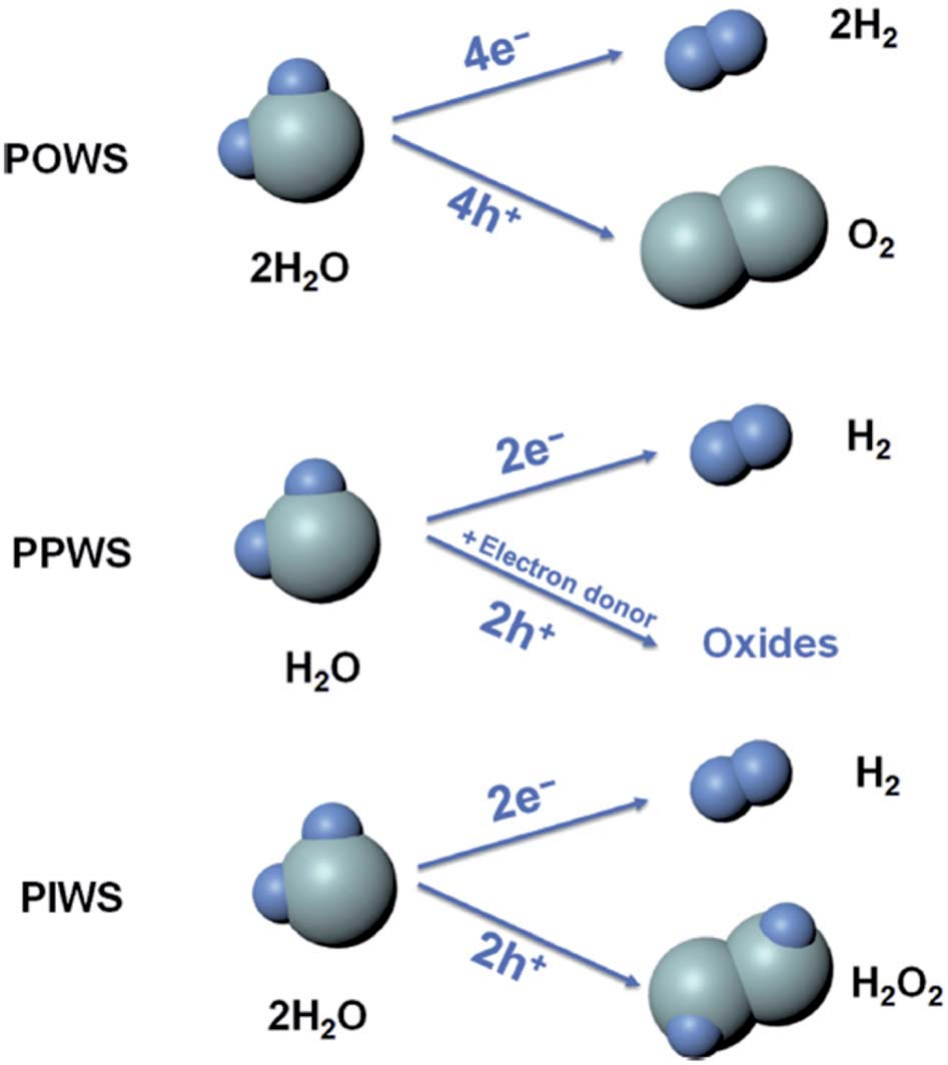
Comparison of the photocatalytic overall water splitting (POWS), photocatalytic partial water splitting (PPWS), and photocatalytic intermediate water splitting (PIWS) processes. This figure has been adapted from ref. 90 with permission from CELL PRESS, copyright 2020.

Among these three approaches, POWS is challenging because of the low photocatalytic efficiency, costly H_2_/O_2_ separation, and reverse reaction mixtures. PPWS requires costly sacrificial agents, and is not a sustainable one although it is commonly used. Furthermore, the photocatalytic rate is quite high. Recently, PIWS became the most challenging one. This approach is helpful for generating the most valuable byproduct H_2_O_2_, and no difficulty is associated with the produced H_2_/O_2_ separation. Furthermore, PIWS provides one H_2_ from two H_2_O molecules (Fig. 13).

Since the discovery of the first MOF for photocatalytic hydrogen production using visible light irradiation, various MOFs with structural diversity as semiconducting materials for hydrogen production have been developed.^91^ As compared to the traditional inorganic semiconductors, semiconducting MOFs have some advantages for use in photocatalytic water splitting reactions. These include the MOF's high porosity, tunable functionality, availability of catalytically active sites, enhanced charge separation ability, and introducing highly light-responsive co-catalyst or functional materials into highly porous MOFs surface. Pristine MOFs have direct uses as catalysts for photocatalytic hydrogen evolution on UV light irradiation. Silva *et al.*^92^ reported on the highly water stable UiO-66 MOF for photocatalytic hydrogen production under monochromatic light irradiation at a wavelength of 370 nm. The photocatalytic activity was carried out in an aqueous solution of methanol, where methanol was used as a sacrificial electron donor. UiO-66 exhibited 2.4 mL H_2_ under 3 h irradiation time. At similar conditions, UiO-66-NH_2_ produced 2.8 mL H_2_ due to the increased light absorption by the amino group present.

**Fig. 14:**
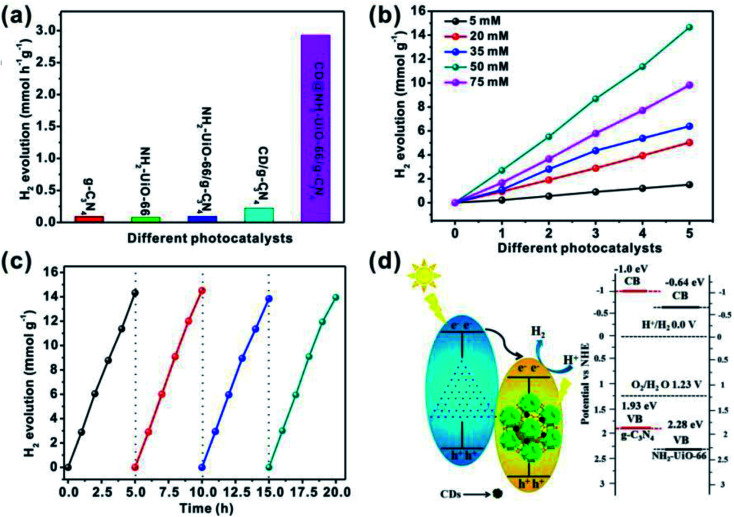
(a) Photocatalytic H_2_ generation rates for g-C_3_N_4_, NH_2_-UiO-66, NH_2_-UiO-66/g-C_3_N_4_, CD/g-C_3_N_4_ and CD@NH_2_-UiO-66/g-C_3_N_4_ under visible light (*λ* >420 nm). (b) NH_2_-UiO-66/g-C_3_N_4_ composites with variable CDs content. (c) The recyclability of the NH_2_-UiO-66/g-C_3_N_4_ composites with 2.77 wt% CDs content for the photocatalytic H_2_ evolution under visible-light irradiation. (d) The proposed mechanism for the photocatalytic process of CD@NH_2_-UiO-66/g-C_3_N_4_. This figure has been adapted from ref. 94 with permission from the AMERICAN CHEMICAL SOCIETY, copyright 2018.

To enhance the photocatalytic activities of a semiconductor, heterojunction construction is an effective route to ensure the charge carriers opposite migration by conduction-band (CB) and valence-band (VB) offsets.^93^ The cocatalyst loading on the semiconducting photocatalyst enhances the photocatalytic H_2_ evolution rate. Zhang *et al.*^94^ reported on the highly stable and visible-light responsive MOF, NH_2_-UiO-66, by constructing an excellent heterojunction with g-C_3_N_4_ and encapsulating CDs into the pores of NH_2_-UiO-66 with 38% Zr in MOF to form a ternary composite photocatalyst, CD@NH_2_-UiO-66/g-C_3_N_4_, as carbon nanodots (CDs) have high light harvesting capacity and excellent electron transfer ability. In this work, the NH_2_-UiO-66/g-C_3_N_4_ heterojunction was built up at first, and the CDs were incorporated into the NH_2_-UiO-66 pores. The CDs were generated from encapsulated glucose in the uniform pores of NH_2_-UiO-66 MOF. The ternary composite, CD@NH_2_-UiO-66/g-C_3_N_4_ exhibited a hydrogen production rate of 2.930 mmol h^−1^ g^−1^ under visible-light irradiation. The rate was quite high as compared to that of the bulk g-C_3_N_4_, NH_2_-UiO-66 and NH_2_-UiO-66/g-C_3_N_4_. The optimum concentration of CDs was 2.77 wt%. The CDs as cocatalysts increase the electron transport properties and cause efficient charge separation. Moreover, CDs in the MOF composite serve as electron transfer mediation to initiate charge separation, improving light absorption and extending the lifetime of photo-induced carriers. This work demonstrates that CDs encapsulation into the pores of MOFs signifies one effective strategy to improve the activity of MOF-based materials (Fig. 14).

**Fig. 15:**
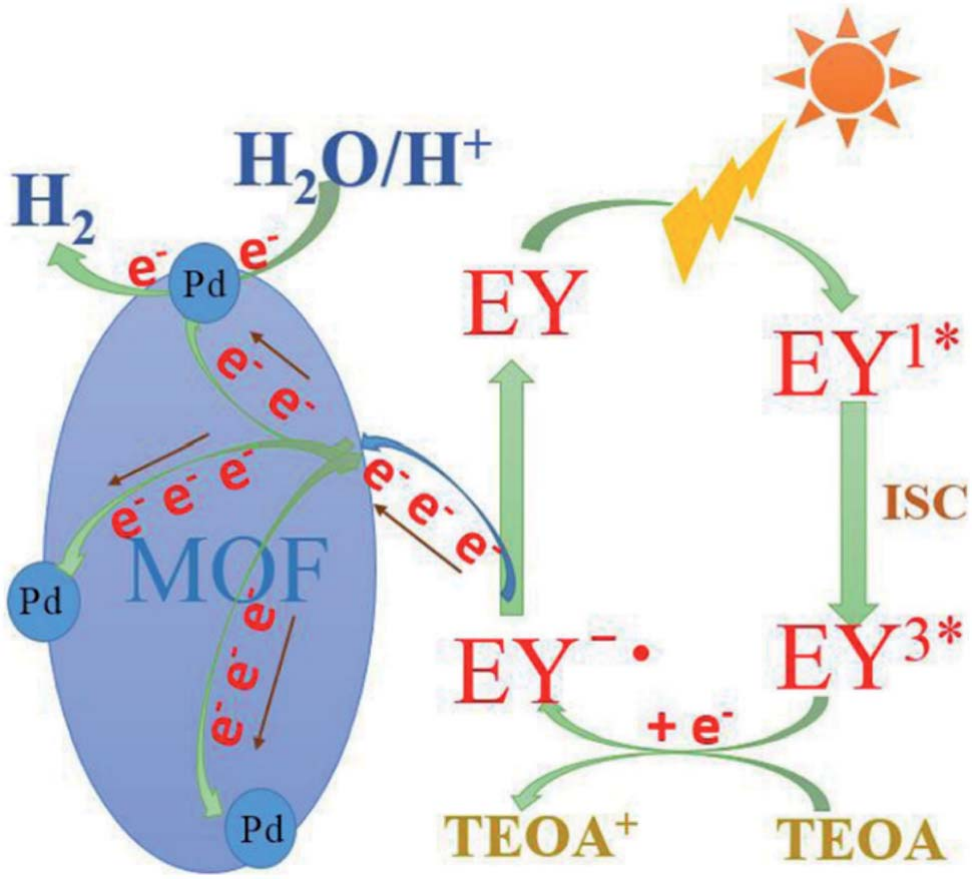
Possible mechanism of the photocatalytic hydrogen evolution in the EY-sensitized system. This figure has been adapted from ref. 95 with permission from SPRINGER SCIENCE + BUSINESS MEDIA, copyright 2017.

Yang *et al.* reported a dye-sensitized Pd/MOF catalyst for photocatalytic hydrogen production.^95^ A nanosized Zr-MOF, UiO-66 (Zr ∼40%) was synthesized solvothermally and Pd was loaded on Zr-MOF by impregnation reduction. The photocatalytic hydrogen evolution study was performed using TEOA as a sacrificial donor in the presence of visible-light (*λ* ≥420 nm), and eosin Y (EY) was introduced as a photosensitizer. The Pd/MOF with Pd loading of 3% exhibited the maximum photocatalytic activity of 2.28 mmol h^−1^ g^−1^. The Pd nanoparticles in the Zr-MOF provided an electronic outlet, and the dye extended the spectral absorption range. The visible light source was a 5 W light-emitting diode lamp of wavelength 420 nm for studying the photocatalytic activity, and the evolved hydrogen was measured by gas chromatography. The mechanism of hydrogen evolution is shown in Fig. 15.

Light-absorbing chromophores such as porphyrin motifs, metal complexes, and NH_2_-BDC can be incorporated into MOFs to enhance the hydrogen evolution rate in the photocatalytic water splitting process, as these chromophore groups in the MOF framework enhance the energy conversion process by facilitating charge separation and improving the light harvesting capacity. Wang *et al.*^96^ reported on the Zr-based MOFs introducing light-harvesting Ir complexes. Pt nanoparticles were introduced into the pores of the MOFs as a co-catalyst, which enhanced the photocatalytic H_2_ evolution activity. The resulting Pt@MOF (Pt/Ir ∼17.8) composite exhibited efficient photocatalytic hydrogen evolution under visible light, with TONs of 7000, which was very high as compared to homogeneous systems.

Hybrid materials of covalent organic frameworks (COFs) and MOFs have drawn great attention for photocatalytic H_2_ production due to their ordered long-range geometry, excellent light absorption capacity, large surface area, and tunable band gaps. Lan and his group introduced a 2-D COF (TpPa-1-COF) with stable MOF (NH_2_-UiO-66).^97^ The MOF was covalently anchored on the surface of COF to form an interesting hybrid of MOF/COF. The resulting porous hybrid material NH_2_-UiO 66/TpPa-1-COF, synthesized in a 4 : 6 ratio exhibited the maximum photocatalytic H_2_ evolution rate of 23.41 mmol g^−1^ h^−1^ with a TOF value of 402.36 h^−1^. The rate was about 20 times greater than that of the parent TpPa-1-COF. The experimental studies and DFT measurement confirmed that COF has effective light absorption capability with similar band gaps in between MOF and COF. There was also an efficient charge separation over the covalent heterojunction interface in the MOF/COF hybrid, which greatly contributed to the reaction rate for H_2_ evolution. The Zr content in the MOF was about 12.72 wt% and co-catalyst Pt loading in COF was 3 wt% for the resulting MOF/COF hybrid for efficient H_2_ production. Naggar *et al.*^98^ reported on two MOF structures containing single and binary central metal ions, such as nickel-benzene dicarboxylic acid (Ni-BDC) and nickel/copper-benzene tri-carboxylic acid (Ni/Cu-BTC). The two MOFs were then used for photocatalytic hydrogen evolution activity, and it was found that Ni-MOF (Ni ∼67% in MOF) exhibited hydrogen productivity of 200 mmol h^−1^ with 50% purity. The Ni/Cu-MOF exhibited hydrogen storage activity due to the involvement of two metal cations within MOF (Table 4).

**Table 4:** A few examples of MOFs-based materials for photocatalytic H_2_ evolution

Sl no	Photocatalyst	Light source	Solvent	Sacrificial agent	H_2_ production rate (μmol g^−1^)	Ref.
1	UiO-66 MOF & UiO-66-NH_2_ MOF	200 W Xe-doped Hg lamp, *λ* ∼370 nm, 3 h	H_2_O/MeOH	—	0.0024 (2.4 mL) & 0.0028 (2.8 mL)	92
2	CD@NH_2_-UiO-66/g-C_3_N_4_	Visible light irradiation (*λ* >420 nm), 5 h	H_2_O	Sodium ascorbate	14 650	94
3	Pd/MOF	5 W light-emitting diode lamp, *λ* ≥420 nm, 4 h	H_2_O	TEOA	9430	95
4	Pt@MOFs	450 W Xe-lamp with a 420 nm cutoff filter, 48 h	H_2_O	TEA	TON ∼ 7000	96
5	NH_2_-UiO 66/TpPa-1-COF	300 W Xe lamp with a cut-off filter of 420 nm, 1 h	H_2_O	Sodium ascorbate	23 410	97
6	Ni-MOF	Visible light lamp, 1 h	H_2_O/MeOH	—	200 000	98

#### (b) Electrocatalytic approach

**Fig. 16:**
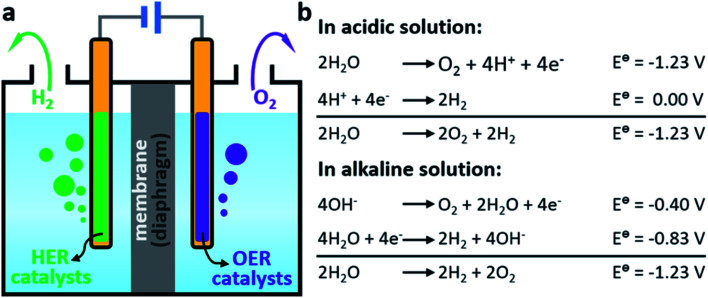
(a) Scheme of conventional water electrolyzers. (b) Water splitting reactions under acidic and alkaline conditions. This figure has been adapted from ref. 109 with permission from the AMERICAN CHEMICAL SOCIETY, copyright 2018.

There are several industrial methods for hydrogen production, such as steam reforming, coal gasification, and water splitting.^99^ Among these, the hydrogen evolution reaction (HER) by water splitting is gaining more importance due to advantages like performing the reaction at RT condition and at ambient pressure, easy separation of the reaction products selectively, and most efficiently, water is the most abundant component on earth. The electrocatalytic water splitting approach for H_2_ production using electrocatalysts was found to be fruitful because of the capability of the electrocatalysts to lower the electric voltage utilization, yielding a high reaction rate. Electrolysis of water mainly involves two half reactions, the hydrogen evolution reaction (HER) and oxygen evolution reaction (OER), where HER occurs at the cathode and OER at the anode of an electroanalyser.^100,101^ The electrochemical HER *via* water splitting process involves two steps: absorption of the formed hydrogen atom during the release of H_2_O or H_3_O^+^ at the catalytically active sites (Volmer reaction) and H_2_ formation and evolution. The details of the HER mechanism by electrocatalytic water splitting process using electrodes under different conditions has been discussed broadly in some previous reports.^102–106^ The electrocatalytic hydrogen evolution reaction mechanism in both acidic and basic media has also been explained in detail in previous reports.^107–109^ Knowing the complete mechanistic insights in both acidic and basic media of electrocatalytic HER would help to differentiate the reaction pathways followed by the electrocatalytic system. Efficient hydrogen production *via* electrolytic water splitting using MOF-based catalysts has become another important strategy to develop renewable fuels as an energy source. Pristine MOFs and MOFs-based materials incorporating a catalytically active moiety into its porous framework can serve as excellent catalysts for electrocatalytic HER (Fig. 16).

Carbon electrocatalysts comprising metal complexes (*e.g.*, MN_*x*_ and MS_*x*_) are considered as promising materials to be used as replacements to costly Pt catalysts for HER. Seifert and Feng *et al.*^110^ reported on carbon-rich 2D MOF-based catalysts incorporating molecular metal dithiolenediamine (MS_2_N_2_, M = Co and Ni), metal bis(dithiolene) (MS_4_), and metal bis(diamine) (MN_4_) complexes, which were used as electrocatalysts for electrocatalytic hydrogen evolution. The 2D MOF-based catalysts with various metal complexes follow the order, MS_2_N_2_ > MN_4_ > MS_4_. The protonation happened on the metal atoms, which was confirmed from experimental and DFT calculation. The simultaneous H_2_ evolution seemed to happen on the M − N units of the MS_2_N_2_ active centers. The electrocatalytic HER performance of the 2D MOF sheets was performed using the rotating disk electrode (RDE) method in the N_2_-saturated aqueous solution. The three different modified electrodes of the 2D MOF-sheets (THTA-Co, THTA-Ni, and THT-Co nanosheets) and a bare electrode were taken in a solution of 0.5 M H_2_SO_4_ for electrocatalytic HER, and it was found that THTA-Co-2D MOF sheets with CoS_2_N_2_ active sites exhibited the best H_2_ evolution performance as compared to the other three. Under a similar current density of 10 mA cm^−2^, THTA-Co-2D MOF exhibited a lower overpotential value (∼92 mV) and lower Tafel slope of 71 mV decade^−1^. At equilibrium potential, the active site (CoS_2_N_2_) of THTA-Co-2D MOF exhibited a lower Gibb's free energy of −0.12 eV than the other MOFs electrodes. The lower Gibb's free energy suggests weaker H* adsorption on the active surface sites and weaker adsorption of H*, leading to excellent catalytic performance for the HER route. The high exchange rate of the current density with the small overpotential value and low Gibb's free energy results in enhanced HER. The order of activity of THTA-Co-2D MOF was found to be CoS_2_N_2_ (−0.12 eV) > CoN_4_ (−0.4 eV) > CoS_4_ (−0.42 eV) for the three active sites.

Loh *et al.*^111^ reported on the GO/copper-MOF hybrid structure as an electrocatalyst for electrocatalytic HER, along with the oxygen evolution and reduction reaction. The catalyst has the ability to coordinate with more electronegative ligands of nitrogen and oxygen functional groups, thereby enhancing the stability of the framework structure, specifically encapsulating GO in acidic medium. Graphene oxide (GO) in the MOF framework serves as linkers to MOF nodes, and also behaves as an electron transfer mediator. The GO-encapsulated Cu-MOF composite with 41% Cu in MOF provides a power density, which is 76% that of the commercial Pt catalyst. The (GO 8 wt%)/Cu-MOF hybrid exhibited the best photocatalytic performance for HER with smaller Tafel slope of 84 mV dec^−1^ with a minimum overpotential of −0.209 V *vs.* RHE at a current density of −30 mA cm^−2^ as compared to that of Cu-MOF and (GO 6 wt%)/Cu-MOF.

Duan and his co-workers reported on an ultrathin nanosheet array of MOFs on various substrates *via* dissolution–crystallization mechanism.^112^ These materials showed excellent performance for HER due to the presence of active molecular metal sites with ultra-small thin nanosheets, enhanced conductivity and ordered porosity. They designed a nickel-iron-based MOF array (NiFe-MOF), which exhibited a minimum overpotential value of 134 mV at a current density of 10 mA cm^−2^ and Tafel slope of 256 mV dec^−1^ for electrocatalytic HER. The presence of 23% Fe impurities in MOF enhanced the activities for Ni-based catalysts by introducing excess structural vacancies. In addition, they demonstrated excellent electrocatalytic performance for OER and overall water splitting.

Roy and his co-workers reported a new MOF comprising cobaloximes that served as metallo-linkers between hexa-nuclear zirconium clusters.^113^ While the reported MOF material, UU-100(Co), was grown on conducting substrates and a reduction potential was applied, the cobaloxime linkers seemed to accelerate electron transport over the thin film. The TON was found to be much higher than the other equivalent cobaloxime system. The UU-100(Co) electrocatalyst with Zr : Co ∼2.8 ± 0.3 showed excellent electrocatalytic H_2_ production from water at acidic medium. The Tafel analysis of the LSV data produced a Tafel slope of 250 mV dec^−1^. When the catalyst was grown on glassy carbon, it exhibited electrochemical HER for 18 h at a constant current density of 1.7 mA cm^−2^. Post-electrolysis analysis confirmed the intact molecular integrity of the cobaloxime linkers in the MOF material (Table 5).

**Table 5:** A few examples of MOFs-based materials for the electrocatalytic H_2_ evolution reaction

Sl. no	Catalyst	Reaction condition	Current density (mA cm^−2^)	Overpotential value	Tafel slope (mV Dec^−1^)	Ref.
1	THTA-Co-2D MOF	0.5 M H_2_SO_4_	10	92 mV	71	110
2	(GO 8 wt%)/Cu-MOF	0.5 M H_2_SO_4_	−30	−0.209 V *vs.* RHE	84	111
3	NiFe-MOF	0.1 M KOH	10	134 mV	256	112
4	UU-100(Co)	NaClO_4_ (0.1 M)/acetate (0.2 M) buffer	1.7	—	250	113

#### (c) Photoelectrocatalytic approach

**Fig. 17:**
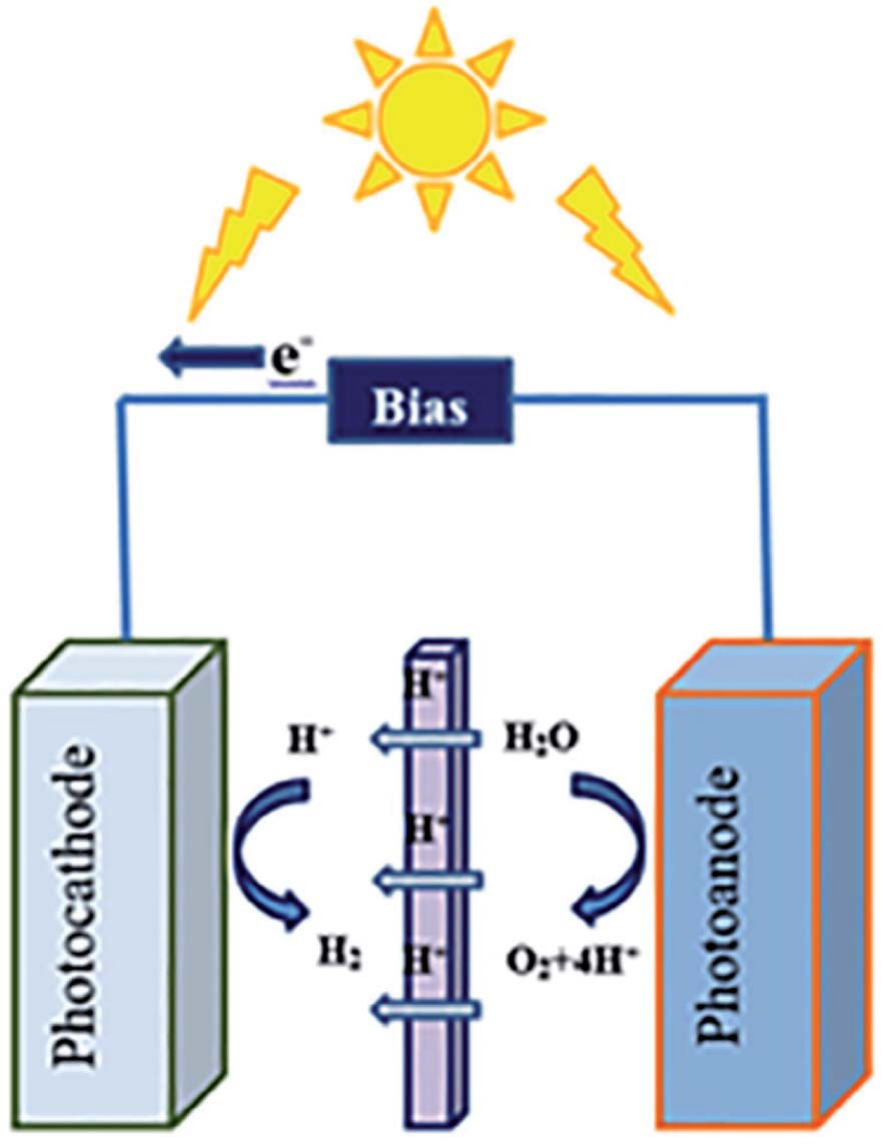
Photoelectrochemical water-splitting cell. This figure has been adapted from ref. 116 with permission from NATURE, copyright 1972.

Photoelectrocatalytic (PEC) water splitting into hydrogen production is a very genuine eco-friendly approach that utilizes solar energy to produce oxygen and hydrogen molecules using a photocatalyst. The PEC water splitting mechanism proceeds through the following steps: (i) water splitting occurs at the photoanode of the electrochemical cell in the presence of solar light, creating photo-generated charge carriers. (ii) At the photoanode, oxygen is produced in the holes created. (iii) External circuits produce electrons at the anode, and these electrons get transferred to the cathode. (iv) H^+^ produced at the anode moves to the cathode through the electrolyte solution to produce H_2_ gas. The net reaction for H_2_ production in PEC *via* water splitting is as shown below (Fig. 17).^114,115^2*hv* + H_2_O (l) = ½O_2_ (g) + H_2_ (g)

For a feasible PEC water splitting reaction, the light energy must possess energy equal to or more than the bandgap of the semiconductor photocatalyst. The first PEC water splitting concept was introduced by Fujishima and Honda in 1972.^116^ Since then, scientists have been trying to develop semiconducting photocatalysts for hydrogen generation by the PEC water splitting approach to convert photon energy of sunlight into electrochemical energy. MOFs with different structural diversity and semiconducting behavior, including the ability to introduce various functional materials into its highly porous surfaces, are drawing much scientific attention in recent years for the PEC hydrogen evolution reaction.

The PEC water splitting activity depends on various factors, such as the bandgap of the semiconductor photocatalyst, sample morphology, and other operating conditions. For efficient catalytic activity, efficient charge separation is a key factor. For efficient charge separation, the band gap of the photocatalyst should be smaller for absorbing a significant amount of solar energy (approx. 1.6–2.2 eV).^117,118^ The particle size also changes the reaction rates. An optimum particle size is very essential for enhanced PEC activity, as the smallest particle size may lead to an enhanced electron–hole pair recombination rate with the close vicinity of charges, decreasing the reaction rates.^119^

Lee *et al.*^120^ reported on the photoactive amine-functionalized Ti-MOF layer (MIL(125)-NH_2_(Ti)), which was uniformly coated on vertically ordered TiO_2_ nanorods (NRs) through the hydrothermal process, and the photoelectrochemical (PEC) water splitting performance of the heterojunction photoanode was studied. MIL(125)-NH_2_/TiO_2_ NRs exhibited a photocurrent density of 1.63 mA cm^−2^ at 1.23 V *vs.* RHE under AM 1.5 G simulated sunlight illumination. The photocurrent density was ∼2.7 times greater than that of pristine TiO_2_ NRs. The water oxidation was enhanced by the efficient photon/electron conversion rate of the MIL(125)-NH_2_/TiO_2_ NRs catalyst at a wavelength of *λ*_ma*x*_ = 340 nm *via* efficient light sorption and charge separation. The MIL(125)-NH_2_ coating enhanced the PEC activity in the TiO_2_ NRs, which has been explained in detail by Lee and his workers in this work. Similarly, Hu *et al.*^121^ reported on the Pt@NH_2_-MIL-125(Ti) catalyst for photoelectrochemical hydrogen production by a double solvent method, which was followed by photoreduction. The synthesized photocatalyst was used as a photocathode material for PEC H_2_ evolution reaction. The 5 wt% Pt loading on MOF increased the photoelectrocatalytic H_2_ production rate. Huang and Wu *et al.*^122^ reported on the p–n heterojunction photoanode based on a p-type porphyrin MOF thin film and an n-type rutile titanium nanorod array for photoelectrochemical water splitting. The TiO_2_@MOF core–shell nanorod array was synthesized by MOF coating on the TiO_2_ nanorod array scaffold by a layer-by-layer self-assembly method. The formed p–n junction between TiO_2_ and MOF improves the extraction of the photogenerated electrons and holes out of the TiO_2_ nanorods. In addition, the MOF coating enhances the efficiency of the charge injection at the photoanode/electrolyte interface. Furthermore, the Co(iii) introduction into the MOF layer improves the charge extraction capacity in the photoanode, thereby enhancing the charge injection efficiency. The resultant photoanode possessed a photocurrent density of 2.93 mA cm^−2^ at 1.23 V *vs.* RHE, which is ∼2.7 times the photocurrent achieved with a bare TiO_2_ nanorod array under the irradiation of an unfiltered 300 W Xe lamp with an output power density of 100 mW cm^−2^. Huo and Liu *et al.*^123^ reported a review on MOFs as photosensitizers for photoelectrochemical water splitting reactions.

## 3. Conclusions and future aspects

Considering the serious global energy demand and extinction of fossil fuels due to daily human activities, it is highly advisable to discover and develop diverse new ways and models to cover up the annihilation gap for a sustained future generation with enhanced fuels and useful chemicals production. In this review, we discussed the recent developments on fuel energy production using MOFs-based materials. We have focused on different fuels production such as hydrocarbons (CO, CH_3_OH, CH_4_, and HCOOH) from green-house gases like CO_2_ and H_2_ production mainly from water splitting reactions, where the employment of different approaches like photocatalytic, electrocatalytic and photo electrocatalytic have been studied. Since the late 1990's until now, MOFs have gained much attentions of the researchers in a non-stoppable way owing to their unique structural properties. The use of MOFs in energy applications with efficient results are growing in the fastest way in recent years. Still, the production of highly efficient fuel sources from MOFs-based materials is restricted to the laboratory scale. Therefore, a fuel production rate in the industrial scale is highly desired. Moreover, CO_2_ reduction into useful materials only in water medium leads to a low conversion rate due to the poor solubility of CO_2_ in water. Researchers have developed a new strategy to overcome this problem using non-aqueous solutions or a mixture of aqueous/non-aqueous solvent, which are costly and not highly abundant. If water can be used as an effective solvent with good catalytic efficiency of the CO_2_ conversion rate to develop standard catalysts, that will be highly appreciable to obtain efficient fuels production. Additionally, CO_2_ conversion into efficient fuels having C_3_ and C_3+_ products are highly challenging these days. Very few C_3_ products from CO_2_ conversion have been reported until now. PEC CO_2_ conversion into fuels like methanol and methane also has been a very challenging approach until now. In addition, the design of excellent MOFs-based catalysts to acquire the efficient production of fuels selectively is highly demanded to boost the world economy. In recent years, many MOFs materials have been utilized for photocatalytic, electrocatalytic and photoelectrocatalytic H_2_ production from water splitting. Still, challenges remain in the design of perfect catalysts with high catalytic efficiency rate, and a proper mechanistic investigation has yet to be covered. The use of different functional materials such as co-catalysts into MOFs enhances the catalytic efficiency of the products formed. Also, sacrificial agents improve the fuel production rate *via* water splitting and CO_2_ conversion. Therefore, the development of MOFs catalysts to produce useful chemicals and fuels with high reaction rates without the assistance of any costly sacrificial materials would be highly considerable. The interactive study of the functional materials with a porous MOFs surface is still not very clear, which requires the attention of the young researchers. Still, so many MOFs and MOFs-based materials with enhanced fuel production rate and selectivity have been explored so far. The unique structural properties and synergistic effect with other functional materials of highly semiconducting MOFs display tremendous activity in various fields, including energy conversion applications and fruitful fuels generation, providing renewable energy resources. Still, there are some limitations for practical applications of MOFs-based materials for fuels and chemicals production *via* CO_2_ conversion and water splitting processes to date. Limitations include the appropriate catalysts and photoreactor design, low conducting properties, low conversion efficiency, lack of chemical stability, electrode potential, large band gap, insufficient catalytically active sites, suitable solvent selection, use of costly sacrificial agents and the large-scale production of fuels and chemicals.

For an efficient energy conversion process using MOF-based catalysts, the catalysts should have appropriate band positions that can be achieved by tuning the MOFs structural functionalities or introducing highly light-responsive guest molecules into MOFs. Solvent selection is another important factor. Proper dissolution of catalysts or reactants into the selected solvent leads to efficient energy conversion processes. The catalysts surface should also possess higher catalytically active sites to achieve high catalytic production rates, which can be obtained by structural/surface modifications of MOFs-based materials. For efficient electrochemical energy conversion, the electrode potential and current densities are two very crucial factors. MOFs-based catalysts also lead to selective chemicals and fuels production, depending on these factors. Most of the known catalysts have typical current densities on the order of mA cm^−2^ of the electrodes. However, in industries, the catalyst surface of the water electrolyzers operates at a magnitude of 2 A cm^−2^. Experimental parameters (such as the temperature, pressure, catalysts concentration, wavelength and duration of illumination, dissolved oxygen concentration) are also important factors for the selective product formation during energy conversion reactions. Suitable co-catalysts loading on MOFs-based catalysts can enhance the catalytic energy conversion rates by changing the energies of the charge transfer process. Additionally, it can enhance the continuous flow of photogenerated charge carriers on the catalysts surface, providing stability to the system.

Limitations also include the use of costly materials that restrict the large-scale production of important fuels and chemicals using MOFs-based catalysts. The costly materials included are mainly costly non-aqueous solvents, sacrificial materials, costly metals, and light sources (Xe, Hg lamp). Synthesizing cost-effective MOFs is very challenging nowadays, and scientists are working on it. To produce cost-effective MOFs, catalyst design should be based on low-cost materials that would additionally exhibit efficient energy conversion. For that, one should try to avoid the use of costly metals and solvents, and using of the easily available natural resources (like water) as a solvent and low-cost reactant materials to produce efficient MOFs-based catalysts with enhanced energy conversion applications. The loading of metal-free semiconducting co-catalysts on the MOFs surface instead of using metals containing costly semiconductors can be an alternative approach for producing cost-effective highly efficient MOFs-based catalysts with reduced toxicity for energy applications. Recently, researchers have found many ways for efficient fuels and chemical production using MOFs-based catalysts. Still, the production of cost-effective MOFs with large scale energy conversions remains a challenge for the upcoming generation to explore in this area.

Briefly, we are focused on the recent highly commanded energy applications of MOF-based materials for fuel and chemical production purpose from CO_2_ conversion and sustainable clean hydrogen production from water. We have mentioned some previous reports related to this topic. We are also trying to show the challenges associated with enhancing the catalytic rate of fuel production, so as to bring this challenge to the focus of researchers. Undoubtedly, MOFs are excellent materials for creating renewable energy resources, but the challenge still remains with dealing with the limitations of the MOFs-based materials and bringing it into practical uses.

## Conflicts of interest

There are no conflicts to declare.

## Supplementary Material
